# Functional connectivity networks for transportation delay analysis: From theory to software

**DOI:** 10.1016/j.trip.2026.102187

**Published:** 2026-09

**Authors:** Carlson Moses Büth, Massimiliano Zanin

**Affiliations:** a Institute for Cross-Disciplinary Physics and Complex Systems (IFISC), CSIC-UIB, 07122, Palma de Mallorca, Spain; b Institute for Cross-Disciplinary Physics and Complex Systems (IFISC), CSIC-UIB, Edifici Complex de Recerca de les Illes Balears, Parc Bit, Palma de Mallorca, 07120, Spain

**Keywords:** Transportation networks, Delays, Functional networks, Causality

## Abstract

Within the endeavour of modelling and understanding the propagation of delays in transportation networks, an approach that has attracted increasing interest in the last decade is the creation of functional network representations. These graphs map elements of interest (e.g. airports or stations) as nodes, and derive pairwise dependency patterns from their dynamics through correlation and causality tests. In spite of multiple notable results, this approach still lacks a coherent framework, with decisions related to many fundamental steps being left to the judgement of the researcher. We here provide an introduction to the theory behind functional networks for transportation systems, detailing the main steps and the associated pitfalls. We further introduce a Python package, *delaynet*, designed to support the researcher in the reconstruction and analysis of such networks. We finally present an analysis of the propagation of delays in the Swiss train system; and discuss future research steps.

## Highlights

• End-to-end pipeline for functional delay networks.
• Directional, lag-aware connectivity with FDR-based pruning.
• Detects bridge nodes and corridors for targeted mitigation.
• Easily track structural change via rolling windows and normalised metrics.
• Applicable across modes; Swiss rail case shows operational value.

## 1 Introduction

Transportation networks are complex systems composed of a large number of interconnected components that facilitate the movement of people and goods, and that have become the backbone of current societies (Cascetta, 2009, Button, 2010). These networks, whether air, rail, road, or maritime, share a common challenge: delays. Delays in transportation systems can propagate through the network, affecting multiple components and leading to cascading failures that significantly impact operational efficiency, passenger experience, and economic outcomes (Carlier et al., 2007, Peterson et al., 2013). Understanding how these delays propagate is crucial for developing effective strategies aimed at their mitigation.

A large body of literature has been devoted to this task, mainly through three complementary approaches. Firstly, one finds the statistical description and modelling of delays (Rebollo and Balakrishnan, 2014, Wang and Vaze, 2016, Kim et al., 2017, Yang et al., 2019, Mitsokapas et al., 2021), in which the shape of the probability distributions (e.g. the presence of long tails and asymmetries) is used to infer potential underlying mechanisms, both for delay generation and propagation. Secondly, the use of regression, data-based, or more recently machine learning and deep learning models (Cannas et al., 2013, Yu et al., 2019, Truong, 2021, Sajan and Kumar, 2021, Lapamonpinyo et al., 2022). These present the advantage of being able to connect causes to effects, hence guiding towards strategies for improving the system; and to further yield tools for forecasting (and hence, hopefully, prevent) delays. Thirdly, one can resort to (usually microscale) simulations, to try to model the relevant aspects of the system under study (Higgins and Kozan, 1998, Barnhart et al., 2014, Wei and Vaze, 2018, Özlem et al., 2021, Zhao et al., 2025b, Xu et al., 2025, Zhao et al., 2025a); in this case, the user can develop what-if scenarios, and thus numerically test the implementation of new mitigation procedures — albeit at a high computational cost. Note that such categorisation is necessarily artificial, and that many studies cross these boundaries — as for instance, any numerical model must be based on real data and statistics. Still, and while these methods have provided valuable insights, they are challenged when the aim is to fully capture the complex dynamics of delay propagation in highly interconnected systems.

In recent years, concepts from statistical physics (Huang, 2009, Reichl, 2016) and complex network theory (Strogatz, 2001, Newman, 2003) have increasingly been applied to transportation systems (Woolley-Meza et al., 2011, Riccardo et al., 2012, Cook et al., 2015, Barthelemy, 2019, Sun and Wandelt, 2021, Olivares and Zanin, 2025). The leitmotif of statistical physics is the study of complex systems, i.e. systems composed of a large number of interacting elements (Anderson, 1972), by inferring microscale laws when only the macroscale dynamics can be observed. The prototypical example is the study of ideal gases: while one cannot observe the position and dynamics of all particles, global laws can still be extracted when observing macroscale properties like pressure and temperature. Similarly, complex networks aim at describing the interactions between the elements of a complex system, usually deriving them from macroscale observables. When applied to transportation systems, these approaches view them as dynamic systems where local perturbations can propagate and amplify through various mechanisms. It is then possible to identify critical nodes, understand systemic vulnerabilities, and finally develop more robust transportation systems.

The statistical physics approach has found extensive application in the study of delays in air transport through the so-called “functional networks”. These were initially developed in neuroscience to model the dynamics of information in the brain (Eguiluz et al., 2005, Power et al., 2010, Park and Friston, 2013), and involve representing air transport as a network. Individual airports are mapped into nodes, and links between pairs of them are created whenever a functional dependency between the corresponding delays is observed, by applying correlation and causality tests (Zanin, 2015, Zanin et al., 2017, Pastorino and Zanin, 2021, Jia et al., 2022, Wang et al., 2022, Feng et al., 2024, Gil-Rodrigo and Zanin, 2024). These dependencies can then be understood as instances of delay propagations, even though confounding effects may also be present. The main advantage of this approach is that only macroscale information is required, e.g. time series representing the average hourly delay at airports. At the same time, it also yields information about how delays may propagate, which airports have a major role in such propagation, or the appearance of constant dependency patterns.

The practitioner wanting to use the functional network approach to study delays, both in air transport and other modes, will face several methodological and computational challenges. To illustrate, while obtaining delay time series is usually simple, these are highly non-stationary (i.e. delays tend to appear with stronger intensity at peak hours), and this may generate spurious correlations and causalities; the solution involves applying one of the main detrending techniques that have been proposed (Olivares et al., 2025). Similarly, many tests can be used to assess dependencies, from correlation-based to causal ones, each yielding different views on the underlying process. Finally, networks have to be processed (by e.g. deleting irrelevant links) and analysed, using one of the many available topological metrics (Costa et al., 2007). Throughout all these steps, choices have to be made, for instance regarding the parameters of a given method, in many cases without theoretical guidelines and subordinate to the researcher’s experience. These problems finally compound due to the lack of unified software libraries, hindering subsequent validations and reproducibility.

In this contribution we review these steps and their pitfalls, explore solutions that have been presented in the literature, and provide the researcher with basic guidelines. This is complemented by presenting *delaynet*, an open-source Python package that aims to solve the aforementioned challenges by combining and systematising time series analysis techniques, network reconstruction methods, and complex network metrics. It offers a unified framework that encompasses the complete analytical pipeline — from data preprocessing to network reconstruction — reducing the risk of methodological inconsistencies that often arise when combining disparate analysis tools, thereby improving the reliability and reproducibility of delay studies. In addition, *delaynet* comes as an off-the-shelf package, flexible to be used for various types of data; thus, while existing research has focused on air transportation (Zanin, 2015, Zanin et al., 2017, Pastorino and Zanin, 2021, Jia et al., 2022, Wang et al., 2022, Feng et al., 2024, Gil-Rodrigo and Zanin, 2024), the implemented methods are applicable to any transportation mode where delay data can be collected as time series.

The software package presented here, and the associated theoretical discussion, are aimed at filling a gap in the literature. Many software pipelines are available to support the reconstruction of functional networks in other fields, mostly in neuroscience (e.g. MNE-Python (Gramfort et al., 2013), NeuroPycon (Meunier et al., 2020), bctpy (LaPlante et al., 2014), dyconnmap (Marimpis et al., 2021), nilearn (Abraham et al., 2014)) and genomics (pySCENIC (Kumar et al., 2021), GReNaDIne (Schmitt et al., 2023), Augusta (Musilova et al., 2024)). The interested researcher can also resort to software packages for general causal inference (Runge et al., 2023, Zhao and Liu, 2023), and for network analysis (Al-Taie and Kadry, 2017). Yet, to the best of our knowledge, none specifically focusing on transportation data has hitherto been proposed, combining all necessary ingredients in a single place. In addition, this approach should not be considered as a complete solution, but rather as one of the many tools available to the practitioner to unveil the underlying dynamics of the system. In other words, functional networks can (and should) be used in conjunction with other techniques, including microscale models and classical statistical analyses, as these are not mutually exclusive. Still, in order for this to be a synergistic process, the researcher should be aware of the underlying hypotheses and limitations of functional networks.

In the remainder of the paper we firstly present a theoretical overview of the main steps in the reconstruction and analysis of delay functional networks, see Section 2. While its content mimics the structure of the *delaynet* package, it is also designed to be an introduction to the topic for the interested researcher, by discussing both fundamental tools and common challenges. Next, Section 3 describes the structure of the package itself, and how the previously mentioned main steps are organised from a software perspective. We then demonstrate the complete workflow on a real dataset in Section 4, a case study of Swiss long-distance rail delays (2022–2025) built from SBB realised timetable data. We finally draw some conclusions and discuss future steps in Section 5.

## 2 Theoretical foundations

**Fig. 1:**
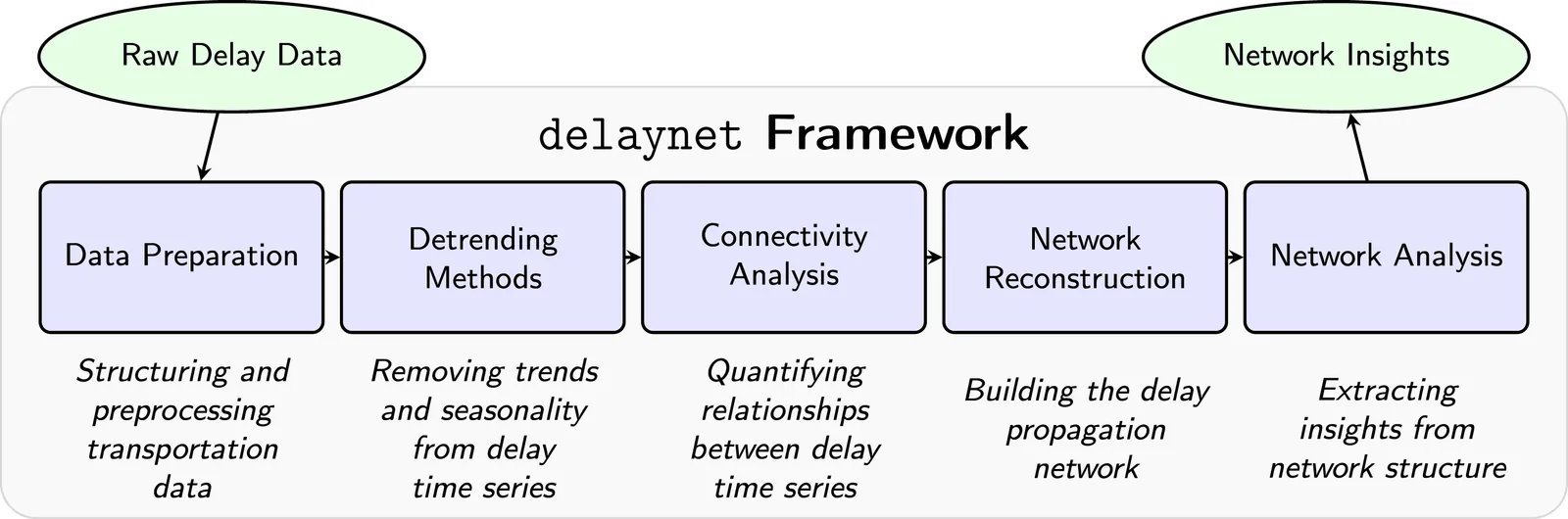
Overview of the main steps in delay functional network analysis, and their implementation in *delaynet*. The framework transforms raw delay data through five sequential stages: (1) data preparation and preprocessing; (2) detrending methods, to remove systematic patterns and seasonality that could mask genuine propagation signals; (3) connectivity analysis, quantifying statistical relationships between delay time series; (4) network reconstruction, which applies thresholding strategies to build functional networks from pairwise connectivity values; and (5) network analysis, to extract structural insights about delay patterns, critical nodes, and system vulnerabilities.

The analysis of delay dynamics in transportation systems using functional networks follows a systematic approach that combines various analytical techniques, integrating contributions from different fields including transportation, information theory and complex network theory. This approach can generally be divided into five fundamental steps: data preparation, detrending methods, connectivity analysis, network reconstruction, and network analysis — see Fig. 1 for a graphical representation. Each component addresses specific challenges in analysing delay data and builds upon the previous steps to provide a complete picture of the delay dynamics.

The following subsections explain each step in detail, focusing on the theoretical foundations, practical considerations and challenges. This structure is also reflected in the internal organisation of *delaynet*, as discussed in Section 3; consequently, what is presented here is both a primer for the interested researcher, and a starting point for the use of the package.

### 2.1 Data preparation

The availability of high-quality and reliable data is the basic requirement behind any data-driven analysis; and, not surprisingly, it also applies to the problem at hand. Functional networks use as starting point a set of time series, each representing the delay observed at the elements of interest (e.g., airports, stations, or route segments) of the system under study. Note that, while previous studies in air transport have focused on airports (Zanin, 2015, Zanin et al., 2017, Pastorino and Zanin, 2021, Jia et al., 2022, Wang et al., 2022, Feng et al., 2024, Gil-Rodrigo and Zanin, 2024), nothing prevents different viewpoints, as e.g. the study of the propagation of delays between sectors. In addition, these time series must be regularly sampled, time aligned, and of uniform length. Crucially, they must have a time resolution consistent with the aim of the analysis — to illustrate, the propagation of delays between airports in the same country cannot be assessed using daily time series, while such low resolution may be suitable in a maritime context. We here also assume that missing values have already been corrected by the user, e.g. through interpolations or forward-filling.

### 2.2 Detrending methods

Raw delay data often contains trends, seasonality, and other systemic patterns that can mask the underlying relationships between components of the transportation network; or, even worse, that may result in the appearance of spurious relationships. Such trend may arise from daily, weekly, or seasonal patterns in transportation demand; long-term growth or decline in usage; or even biases in delay reporting. If not properly addressed, these trends can lead to spurious correlations and misleading results in connectivity analyses.

To illustrate this point, imagine the case of two independent airports, whose traffic peaks respectively at 13:00 and 14:00 UTC. It is reasonable to assume that delays will also peak at those times, as the resources of the airports may be overexerted. If one then calculates a correlation between both airports’ time series of delays, this will yield a propagation between the former and the latter, even though such propagation does not exist; and similar results may be obtained with other causality metrics. In mathematical terms, any measure averaged over a non-stationary time series will not be accurate; stationarity is a prerequisite for standard tests (including the t- and F-tests) and models (like the Auto Regression Moving Average, ARMA).

Multiple detrending methods that can be applied to transportation delay time series have hitherto been proposed, each with its own strengths and limitations. Comparisons using synthetic and real-world time series are also available, see for instance Olivares et al. (2025). For the sake of completeness, we here describe the main ones:

• **Identity transformation.** Simple copy of the original time series, i.e. no detrending is performed. Not to be used in real analyses, it is nevertheless an important method for testing purposes, e.g. as a baseline against which other methods can be compared.
• **Delta Detrending.** This method performs local mean subtraction by removing the average of a window of values centred around each point:
  (1) $$x^{\prime }\left(t\right)=x\left(t\right)-\frac{1}{2w+1}\overset{t+w}{\underset{k=t-w}{\sum }}x\left(k\right),$$
  where w is the window size. Delta detrending is particularly effective at removing local trends while preserving short-term fluctuations that might indicate delay propagation. The choice of window size is critical: too small a window might not capture the trend effectively, while too large a window might remove important signals.
• **Second-Order Differentiation.** This method applies the difference operator twice to remove both constant and linear trends (Kirchgässner et al., 2013):
  (2) $$x^{\prime }\left(t\right)=\left[x\left(t\right)-x\left(t-1\right)\right]-\left[x\left(t-1\right)-x\left(t-2\right)\right]=x\left(t\right)-2x\left(t-1\right)+x\left(t-2\right).$$
  Second difference detrending is particularly useful for transportation delay data that exhibits linear growth or decline over time.
• **Z-Score.** This method normalises the data by subtracting the mean and dividing by the standard deviation, often calculated within specific time windows or periods:
  (3) $$x^{\prime }\left(t\right)=\frac{x\left(t\right)-\mu_{p}}{\sigma_{p}},$$
  where $\mu_{p}$ and $\sigma_{p}$ are the mean and standard deviation for the period p to which time point t belongs. Z-Score detrending is effective for removing periodic patterns, such as daily or weekly cycles in transportation delays.

### 2.3 Connectivity analysis

After detrending, the next step in reconstructing functional networks is to quantify the relationships between delay time series of the different components of the transportation network. In other words, we aim at detecting and measuring how delays in one part of the system influence delays in another one, accounting for the time it takes for these influences to propagate. Given two time series, such analysis is done by applying different metrics and tests to them; this process is eventually repeated for all pairs of time series, to obtain a network view.

In the literature, many alternative metrics have been used, mostly adapted from the solutions common in brain functional network reconstruction. The output in all cases is the same, and comprises both the p-value of the metric and the best lag — i.e. the statistical significance and the time difference of the strongest observed relationship. Starting from the linear ones, these include:

• **Linear Correlation (LC).** The Pearson’s correlation coefficient measures the linear relationship between two time series at a lag τ:
  (4) $$r_{\tau }=\frac{{\mathrm{cov}}_{xy}}{\sigma_{x}\sigma_{y}}=\frac{\overset{T}{\underset{t=\tau +1}{\sum }}\left[x\left(t-\tau \right)-\overset{¯}{x}\right]\left[y\left(t\right)-\overset{¯}{y}\right]}{\sqrt{\overset{T}{\underset{t=\tau +1}{\sum }}{\left[x\left(t-\tau \right)-\overset{¯}{x}\right]}^{2}}\sqrt{\overset{T}{\underset{t=\tau +1}{\sum }}{\left[y\left(t\right)-\overset{¯}{y}\right]}^{2}}},$$
  where $\overset{¯}{x}$ and $\overset{¯}{y}$ are the sample means, and $\sigma_{x}$ and $\sigma_{y}$ are the sample standard deviations of x and y, respectively. ${\mathrm{cov}}_{xy}$ is the sample covariance of x and y.
• **Rank Correlation (RC).** The Spearman’s rank correlation uses the ranks of the values rather than the values themselves, making it more robust to outliers and nonlinear monotonic relationships:
  (5) $$\rho_{\tau }=\frac{\mathrm{cov}\left(R_{x},R_{y}\right)}{\sigma_{R_{x}}\sigma_{R_{y}}},$$
  where $R_{x}$ and $R_{y}$ are respectively the ranks of x(t) and y(t).
• **Granger Causality (GC).** This approach tests whether past values of one time series provide statistically significant information about future values of another time series, beyond what is explained by the past values of the latter (Granger, 1969, Diebold, 1997, Kirchgässner et al., 2013). In the context of transportation networks, GC examines whether historical delays at a source node help predict future delays at a destination node, after accounting for the destination’s own delay history. The method works by comparing two linear models, usually Ordinary Least Squares ones: a restricted model that predicts future delays at the destination using only its own past, and an unrestricted model that incorporates past delays from both the destination and potential source nodes. If including the source node’s delay history significantly improves prediction accuracy, we can infer that delays propagate from the source to the destination. In practice, the implementation tests multiple time lags to identify the optimal propagation time between nodes. For each potential lag, the algorithm constructs appropriate lag matrices, fits both restricted and unrestricted regression models, and compares their predictive performance. The lag that yields the strongest statistical evidence for delay propagation is selected as the optimal propagation time between the two components. Since its inception, the GC test has found wide-ranging applications in various fields such as economics, engineering, sociology, biology, and neuroscience (Freeman, 1983, Reuveny and Kang, 1996, Ding et al., 2006, Shojaie and Fox, 2022). Limitations have also been highlighted, like the sensitivity to confounding effects (Grassmann, 2020), the failure to detect non-linear relationships (Maziarz, 2015), or the inability to handle missing or irregular data (Zanin, 2021).

These linear metrics present the advantage of being simple, both in the computation and the interpretation of the results. On the other hand, their linear nature implies that only the linear part of the propagation is described, and they may therefore understate, or completely miss, more complex dependencies. The practitioner may then want to resort to non-linear tests, including:

• **Mutual Information (MI).** This information-theoretic measure quantifies the amount of information shared between two time series X and Y (Büth et al., 2025):
  (6) $$I\left(X;Y\right)=\underset{x\in X}{\sum }\underset{y\in Y}{\sum }p\left(x,y\right)\log\frac{p\left(x,y\right)}{p\left(x\right)p\left(y\right)}.$$
  Mutual information can capture both linear and nonlinear relationships, but crucially does not indicate directionality, i.e. I(X;Y)=I(Y;X).
• **Transfer Entropy (TE).** This measure quantifies the directed transfer of information from one time series X to a second one Y, by measuring how the uncertainty in the latter is reduced by the former (Schreiber, 2000, Büth et al., 2025):
  (7) $$T_{X\to Y}=\sum p\left(y_{t+1},y_{t}^{\left(k\right)},x_{t}^{\left(l\right)}\right)\log\frac{p\left(y_{t+1}|y_{t}^{\left(k\right)},x_{t}^{\left(l\right)}\right)}{p\left(y_{t+1}|y_{t}^{\left(k\right)}\right)},$$
  where $y_{t}^{\left(k\right)}$ and $x_{t}^{\left(l\right)}$ represent the past k and l values of Y and X respectively. Transfer entropy can detect nonlinear causal relationships, being a generalisation of the GC (Barnett et al., 2009); and is thus particularly useful in the context at hand.
• **Continuous Ordinal Patterns (COP).** This approach generalises traditional ordinal pattern analysis (Bandt and Pompe, 2002) by evaluating time series in terms of their distance to continuously defined patterns (Zanin, 2023):
  (8) $$\phi_{X}\left(t\right)=\frac{1}{2D}\overset{D-1}{\underset{i=0}{\sum }}\left|\pi_{i}-\mathrm{norm}\left(x_{t+i}\right)\right|.$$
  In the previous equation, π represents a predefined continuous pattern of length D, the latter also known as the embedding dimension; and norm(⋅) is the operator normalising a segment of the time series to values between −1 and 1. ϕ(t) can then be seen as a transformed version of the original time series, representing the evolution through time of the relevance of the pattern π. Given two input time series X and Y, the causality between them can be evaluated by firstly applying the same pattern π to both of them, thus obtaining two time series $\phi_{X}\left(t\right)$ and $\phi_{Y}\left(t\right)$; for then calculating the GC test between them (Zanin, 2024). In other words, π is used to extract what makes the two time series unique, and the GC test is used to detect whether the unique dynamics of the two elements are causally connected. Due to the non-linear nature of the transformation induced by π, this approach allows to efficiently detect non-linear causality relations without the need of a priori assumptions.
  To identify the most informative pattern for connectivity analysis, COP generates multiple random patterns and evaluates their discriminative power. For each candidate pattern, the method calculates the statistical difference between the pattern-transformed original time series and a shuffled version, selecting the pattern that maximises this difference using the Kolmogorov–Smirnov test. This pattern selection process ensures that the chosen transformation captures genuine temporal structures rather than random fluctuations.

#### 2.3.1 Statistical significance and interpretation

To determine whether a detected connection is statistically significant, it is customary to rely on the p-values yielded by the connectivity measures; these are obtained by comparing the obtained metric to what is expected under a null hypothesis of no connection. Some measures (e.g. correlations, and the GC and COP tests) naturally yield such p-value; in all other cases, an approximation is obtained either by random permutations or bootstrapping, as selected by the user.

**Fig. 2:**
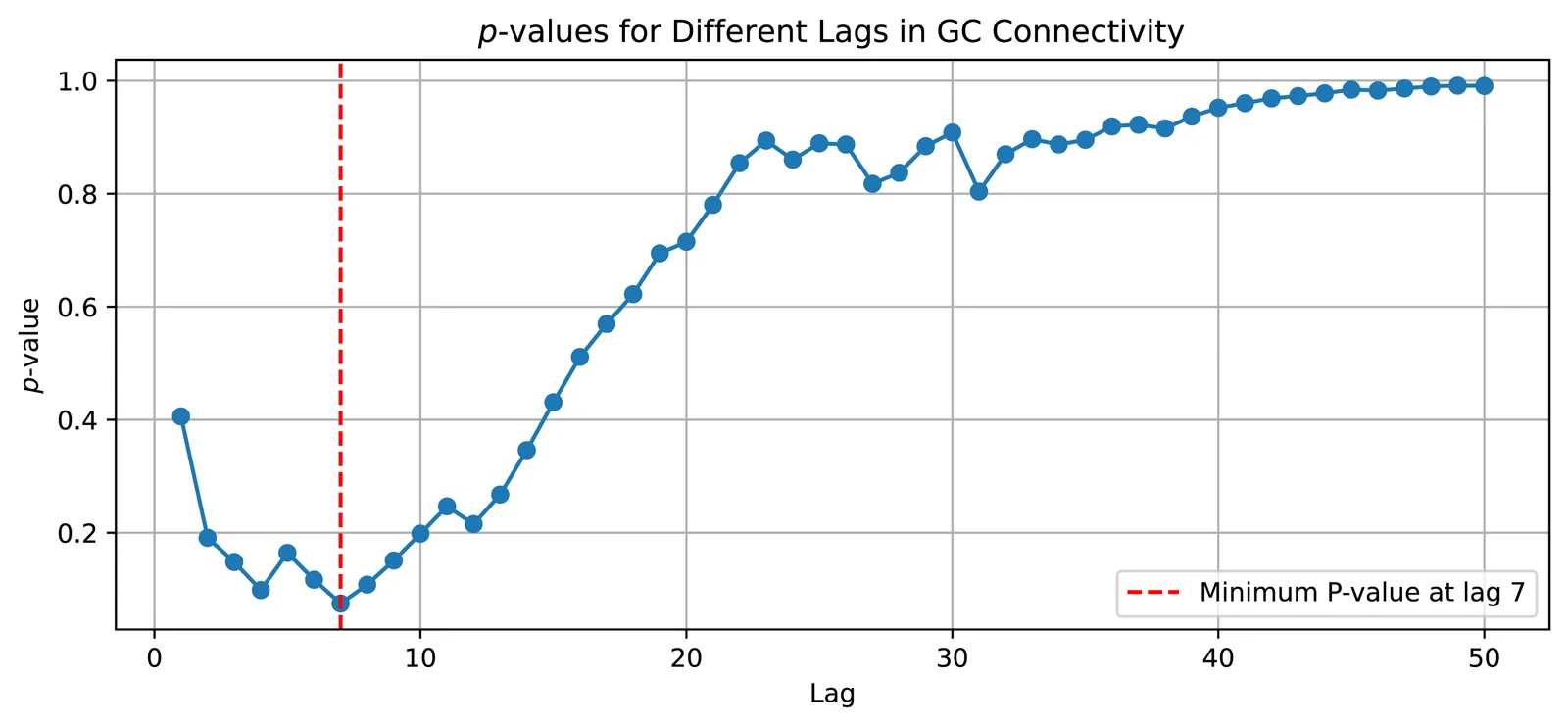
Determination of optimal lag using p-values in a Granger Causality analysis between two synthetic time series. The vertical red dashed line indicates the minimum p-value obtained. See main text for details.

All measures further include a lag, representing the expected time of the functional relationship. This is usually optimised by testing different lag values, to identify the optimal delay that produces the smallest p-value. This is illustrated in Fig. 2, which shows the evolution of the p-values calculated for each lag from 1 to 50 time steps, as obtained by the GC on two synthetic time series created with a delayed causal network (see Section 2.1). The minimum p-value (at lag 7) is interpreted as the optimal delay, indicating the most significant causal connection. Note how p-values increase for high values of the lag; as their calculation requires more complex models, the corresponding degrees of freedom also increase, resulting in less statistically significant results.

As a final note, it is important to highlight the meaning of the patterns identified through the aforementioned tests. These may prima facie be understood as propagation instances, i.e. flows of delays between two airports, in a way similar to how these links are commonly described as flow of information in functional brain networks. Yet, this is only true if no confounding effects are present, i.e. sources of delays not considered in the evaluation of the link and that can affect both nodes at the same time. These confounding effects may also stem from the presence of non-stationarities in the data, which could remain after the detrending process described above. Even when these are not present, it is important to note that measures called “causal”, as e.g. the Granger Causality, only measure predictive causality, i.e. how the delay of one node helps predict the future evolution at a second one; in contrast, a true causality also requires the possibility of making changes in the system through intervention and counterfactual checks (Granger, 1988, Bareinboim et al., 2022). In short, the researcher should be aware of the limitations of the approach, and consider them in the interpretation of the results.

#### 2.3.2 Comparing connectivity measures

One may intuitively expect the existence of a hierarchy between the connectivity measures described above; for instance, the Transfer Entropy is a much more sophisticated metric than a simple linear correlation, and hence should generally be preferred. Reality is nevertheless more complex. Firstly, non-linear approaches usually require larger quantities of data (i.e. longer time series) to detect relationships; hence they may not be suitable when fast dynamics ought to be analysed, e.g. what happened in a specific day or week. Secondly, when the propagation under analysis is mainly linear, non-linear approaches may be ill-suited to describe them. Thirdly, one has to consider that linear approaches do not necessarily disregard non-linear relationships; more correctly, they are only able to describe their linear part, or to generate a linear approximation of them.

**Fig. 3:**
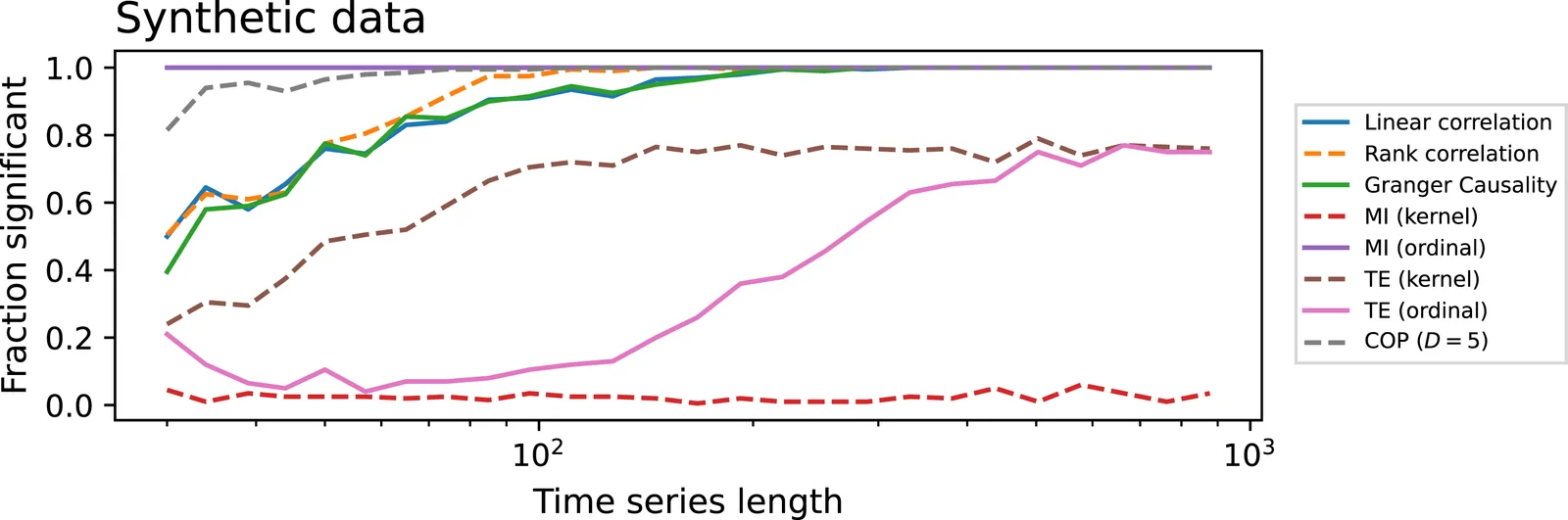
Comparison of different connectivity measures as a function of the time series length, on synthetic data generated using the delayed causal networks (see Section 2.1). See the legend for a list of measures, with the corresponding estimator within parentheses. Results correspond to 200 randomly generated time series.

To illustrate this point, we considered the delayed causal network model described in Section 2.1, and generated time series of variable length with two nodes randomly connected between them; Fig. 3 then represents the fraction of times each measure was able to detect the propagation (using a significance level α=0.01), as a function of the time series length. It can be appreciated that linear approaches (linear correlation, rank correlation and Granger Causality) generally require shorter time series to achieve good results. Non-linear approaches can also work well, but their performance is highly dependent on the underlying estimator. To illustrate, MI detects the relationship when using an ordinal estimator, but not with a kernel one; yet, the opposite occurs for the TE.

**Fig. 4:**
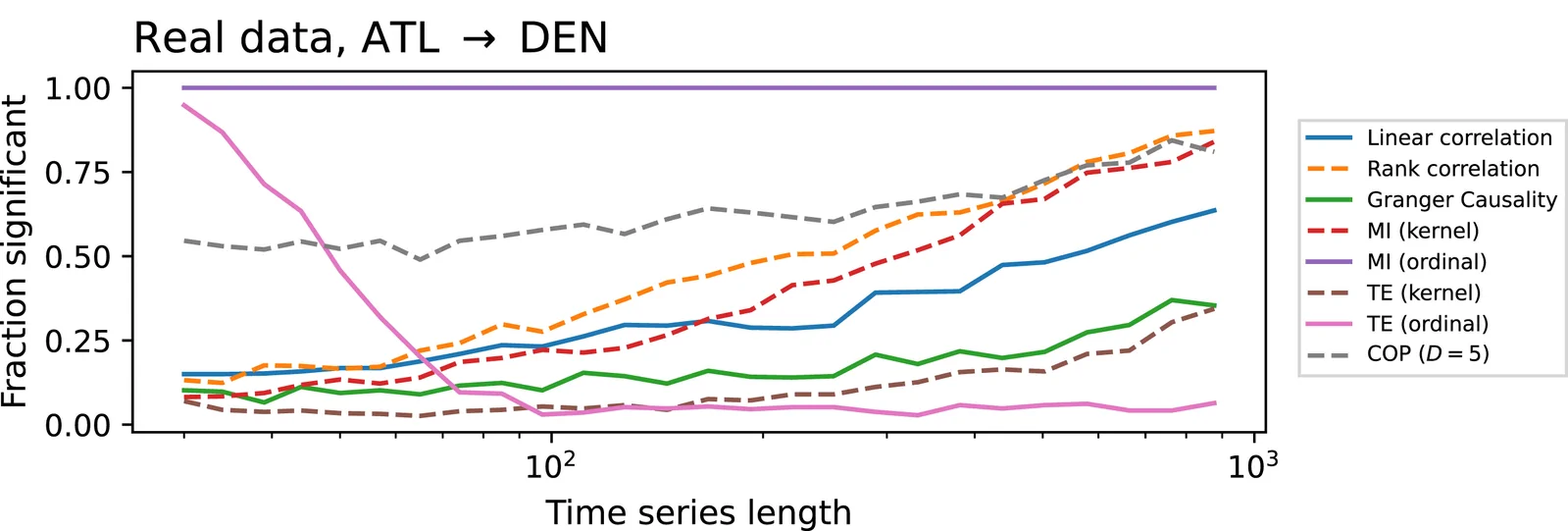
Comparison of different connectivity measures as a function of the time series length, on real data for the Atlanta (ATL) and Denver (DEN) airports. See the legend for a list of measures, with the corresponding estimator within parentheses. Results correspond to 500 randomly extracted time series.

We repeated the same analysis using real delay data for the Hartsfield-Jackson Atlanta International Airport (ATL) and the Denver International Airport (DEN). Delays of landing flights between years 2015 and 2019 have been obtained from the Reporting Carrier On-Time Performance database of the Bureau of Transportation Statistics, U.S. Department of Transportation, freely accessible at https://www.transtats.bts.gov. Time series have been detrended using a Z-Score approach with a period of 24 h; and sub-windows of different lengths have randomly been extracted. Fig. 4 then reports the results in the same format as Fig. 3 above.

As ought to be expected, the results with real data are more complex to interpret. On the one hand, linear approaches underperform when compared to what obtained in Fig. 3; for instance, both GC and LC barely reach 50% of significant fractions. This is possibly due to the non-linear nature of the underlying relationships, which in turn requires longer time series to be correctly estimated. It is also noteworthy the behaviour of the TE with ordinal estimator, which yields good results only for short time series. This may be due to the presence of short-term and non-linear patterns, which are nevertheless lost (or diluted) when considering multiple weeks of data.

In short, the practitioner must be aware that “one size does not fit all”: different connectivity measures can reveal different patterns of delays, at the same time having different requirements in terms of data volumes. This highlights the importance of using multiple complementary approaches to get a complete understanding of the system’s dynamics.

### 2.4 Network reconstruction

Once connectivity measures have been calculated between all pairs of components in the transportation system, the next step is to reconstruct the functional delay network. This network represents how delays are connected throughout the system, with nodes representing transportation components, e.g. airports or train stations, and edges representing the relationships between them. In a simplified interpretation, these edges would be interpreted as propagation patterns, although the limitations described above apply.

The output is twofold. On the one hand, it includes a weight matrix W, where each element $w_{ij}$ reports the p-value between components i and j. Note that self-connections are usually discarded, i.e. $w_{ij}=1.0$ for i=j. On the other hand, it also includes a lag matrix L, where each element $l_{ij}$ represents the time lag at which the strongest connection was found, as illustrated in Section 2.3.1. This last matrix can be used to describe the time scale of the underlying relationship or propagation, and hence to discriminate between direct and potentially indirect ones — see for instance Wang et al., 2022, Pastorino and Zanin, 2023.

The framework reconstructs networks that are directed by design, i.e. $w_{ij}\neq w_{ji}$, reflecting the inherently directional nature of delay propagation. This is naturally obtained in the case of causality tests (including GC and TE), where such directionality is inherent to their definition. In contrast, similar results are obtained by shifting time series in time for correlation metrics (LC, RC and MI). To illustrate, delays at node i affecting node j after τ hours represent a fundamentally different relationship than delays at node j affecting node i after a similar number of hours.

In order to discard those relationships that are not statistically significant, two alternatives can be employed:

• **Statistical Thresholding.** This approach uses a significance level (e.g., α=0.05 or α=0.01) to determine which connections to include:
  (9) $$A_{ij}=\left\{\begin{array}{cc} 1\; & \text{if}w_{ij}<\alpha \\ 0\; & \text{otherwise} \end{array}\right)$$
  Where A is the resulting binary adjacency matrix of the network. This approach ensures that only statistically significant connections are included, but it does not account for multiple testing issues.
• **False Discovery Rate (FDR) Control.** This method adjusts the threshold to control the expected proportion of false positives among all detected connections:
  (10) $$A_{ij}=\left\{\begin{array}{cc} 1\; & \text{if}w_{ij}<\alpha_{\mathrm{FDR}}\\ 0\; & \text{otherwise} \end{array}\right)$$
  $\alpha_{\mathrm{FDR}}$ is determined using procedures like the Benjamini–Hochberg method. FDR control is particularly important when analysing large transportation networks with many potential connections.

#### 2.4.1 Example of reconstructed networks

**Fig. 5:**
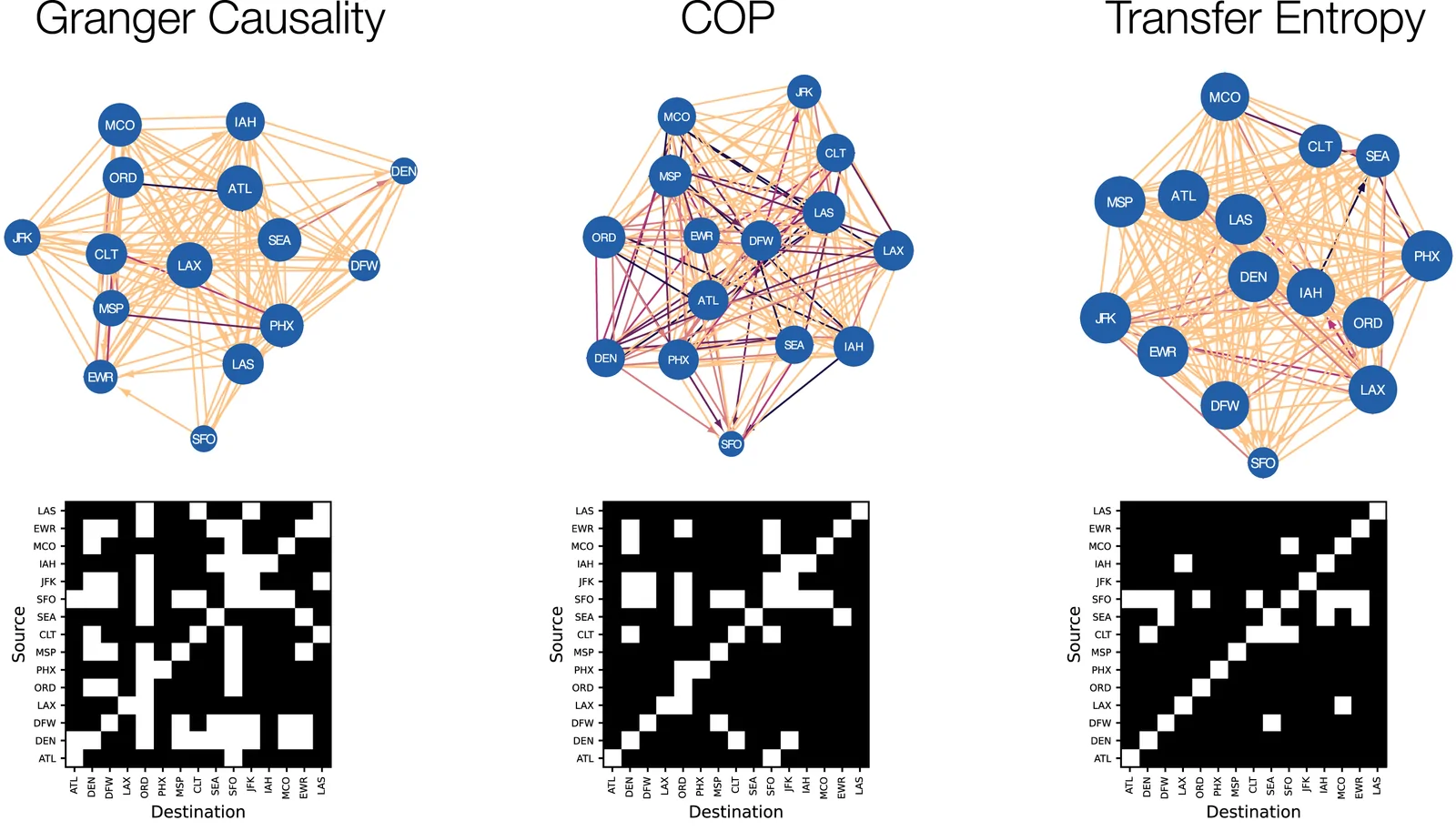
Functional networks corresponding to the top-15 US airports, using data for year 2019. Left, centre and right networks have been reconstructed using respectively Granger Causality, COP, and Transfer Entropy (with a box kernel). The top pictures depict the network, with node sizes proportional to the out-degree, and the colour of link indicating the optimal lag (from 1 h, light shade, to 6 h, dark shade). The bottom graphs report the corresponding adjacency matrices; black squares indicate the presence of a causal connection between the corresponding airports, white squares the absence thereof.

As an example of the reconstruction of adjacency matrices, we consider again the data of the Bureau of Transportation Statistics, this time for the top-15 airports (in terms of the total number of operations) and for year 2019. From it, we extracted time series of average hourly landing delays, and detrended them using the Z-Score approach (with a periodicity of 24 h). We finally reconstructed the adjacency matrices using three different connectivity measures, and filtered the network using a Bonferroni correction for α=0.01. The resulting networks and adjacency matrices are depicted in Fig. 5, for Granger Causality (left), COP (centre), and Transfer Entropy (right).

Several interesting facts can be observed. First of all, all three networks are highly connected. This is to be expected, as we only considered very large airports that are highly interconnected, and that can easily spread delays between them — see Li and Jing (2021) for a discussion on the role of large airports in the US, and especially for the correlation between size of an airport and its importance for the propagation. Secondly, the networks reconstructed with the COP and TE measures are denser than the one obtained by the GC; this may indicate that many relationships have a non-linear nature, which the latter test is ill-designed to detect. Thirdly, the GC and TE seem to mostly detect relationships in short time scales; larger lags (represented by dark links) are more frequent in the COP network.

A word of caution should be raised regarding the high number of propagation instances with lags equal to one hour, i.e. on time scales shorter than the duration of the corresponding flight. Several reasons may be behind this. On the one hand, airlines may adapt their operations using information about the delay of flights that have yet to land, for instance deciding not to recover a delay when a connecting flight is also known to be delayed — something known as Dynamic Cost Indexing (Cook et al., 2009, Delgado et al., 2016). Notably, this implies that what is here measured is the propagation of information about delays, and not delays themselves; a fact that may even create paradoxical situations, like propagations going backwards in time (Wang et al., 2022, Pastorino and Zanin, 2023). On the other hand, such short propagation instances may be due to disruptions happening concurrently at several airports, as e.g. global adverse weather conditions; or, in other words, to the presence of confounding effects. This can be confirmed by repeating the analysis over shorter time windows, ideally in periods in which no such events occurred; and can be solved by deleting propagation links that are physically unfeasible. In short, this simple example illustrates the importance of a correct measure selection; the need of carefully inspecting the results for soundness; and that caution should be exercised in interpreting relationships as propagation instances.

#### 2.4.2 On the interpretation of p-values

As previously detailed and further illustrated in Fig. 5, the result of the analysis in an unweighted network, i.e. each link either exists or does not — in other words, each element $A_{ij}$ of the adjacency matrix can only be zero or one. While alternatives are available, these have to be evaluated with caution under the light of the output of the analysis.

It is firstly tempting to consider the obtained p-value as a metric of the strength of the relationship. One should nevertheless recall that the p-value of a test only represents the statistical significance of results, i.e. how frequently a relation of the same (or larger) magnitude is observed under the null hypothesis; to illustrate, in the case of the GC, it is assessing whether the causing time series is reducing the error in the forecast of the caused one in a significant way. It is therefore only a way to accept or reject the presence of a relationship (here, of a delay propagation). Again in the case of the GC, a smaller p-value indicates that the prediction is better, but this is also affected by aspects like the data size and their probability distribution. For instance, consider two time series of length 1000, x(t)=N(0,1) and y(t)=x(t−1)+N(0,1), with N representing numbers drawn from a normal distribution. Due to the delayed coupling, a GC test correctly detects the presence of a causality x→y with a median p-value of $\approx 10^{-150}$. Now let us increase the standard deviation of the second time series, i.e. y(t)=x(t−1)+N(0,2); the resulting median p-value drops to $\approx 10^{-50}$, even though the intensity of the coupling is exactly the same. In short, interpreting a p-value as a proxy of the intensity of the relationship can only be done *caeteris paribus*, i.e. everything else being equal.

Secondly, it is customary to apply a small threshold for the statistical significance, e.g. α=0.05 or 0.01, as this ensures a minimal number of false positives (i.e. instances in which the measure detects a relationship that is actually not there). Yet, this also implies that some true propagation links are deleted by the filtering — i.e. false negatives may appear. A more truthful representation of the system may be obtained by balancing the number of true and false positives, for instance by increasing the value of α. To the best of our knowledge, this has only been studied once (Acharya et al., 2024), and no strategies for setting the best α in this context have hitherto been proposed.

### 2.5 Network analysis

Once the functional networks have been reconstructed, the literature provides a large number of topological metrics to characterise them, i.e. measures describing specific properties or structural features (Costa et al., 2007). These metrics help to identify critical components, understand global connectivity patterns, and inform strategies for improving system resilience. For the sake of clarity, we here describe some foundational ones, organised in families in what follows.

#### 2.5.1 Macroscale properties

• **Link Density.** The proportion of possible connections that are actually present in the network:
  (11) $$D=\frac{\left|E\right|}{\left|V\right|\left(\left|V\right|-1\right)},$$
  where |E| is the number of edges and |V| is the number of nodes. For directed networks, this represents the ratio of existing connections to the maximum possible number of connections, i.e. n(n−1). A high density indicates extensive relationships throughout the system, hence more chances for interdependencies.
• **Global Efficiency.** This metric quantifies how efficiently information can propagate through the network, calculated as the average of the inverse of the shortest path length between all pairs of nodes (Latora and Marchiori, 2001):
  (12) $$E_{glob}=\frac{1}{\left|V\right|\left(\left|V\right|-1\right)}\underset{i\neq j}{\sum }\frac{1}{d_{ij}},$$
  where $d_{ij}$ is the shortest path length between nodes i and j. Values range from 0, completely disconnected, to 1, perfectly efficient. Higher values imply that a delay generated in one part of the network can easily affect other regions.
• **Transitivity.** Also known as the global clustering coefficient, transitivity measures the tendency of the network to form clusters, calculated as the fraction of all possible triangles present in the graph:
  (13) $$T=3\frac{\text{number of triangles}}{\text{number of triads}}.$$
  Triangles here refer to groups of three nodes fully connected between them, while triads are sets of three nodes with at least two edges between them. Hence, high values of the transitivity (i.e. close to one) indicate that the neighbours of a node tend to be connected to each other. In the context of delay networks, high transitivity may indicate the presence of regional clusters, or of groups of elements that tightly share delays. Note that for directed networks, the direction of edges is usually ignored when calculating transitivity, as the concept of triangles is not uniquely defined in these networks.
• **Reciprocity.** This metric measures the tendency of pairs of nodes to form mutual connections in a directed network; or, in other words, whether a delay pattern a→b tends to be associated to an opposite pattern b→a. It is defined as the fraction of edges that are reciprocated:
  (14) $$R=\frac{1}{\left|E\right|}\underset{i,j}{\sum }A_{i,j}A_{j,i}=\frac{1}{\left|E\right|}\mathrm{Tr}\;A^{2},$$
  with A being the adjacency matrix of the graph. Note that $A_{i,j}A_{j,i}=1$ if and only if i connects to j and vice versa. Reciprocity is only defined for directed networks, as in undirected networks all connections are reciprocal by definition.

#### 2.5.2 Centrality measures

Centrality measures aim at identifying the most important nodes in the network, or, in other words, to assign a relative importance to each node, which can then support their ranking. Note that this is an ill-defined concept, as importance can have different interpretations in different contexts; as a consequence, a large number of complementary centrality measures have been proposed in the past (Batool and Niazi, 2014, Grando et al., 2016, Saxena and Iyengar, 2020). *delaynet* includes the following ones:

• **Betweenness Centrality.** This measure quantifies the extent to which a node lies within the shortest paths between other pairs of nodes (Freeman, 1977, Brandes, 2008):
  (15) $$C_{B}\left(i\right)=\underset{s\neq i\neq t}{\sum }\frac{\sigma_{st}\left(i\right)}{\sigma_{st}}.$$
  Here, $\sigma_{st}$ is the number of shortest paths from node s to node t, and $\sigma_{st}\left(i\right)$ is the number of those paths that pass through node i. Nodes with high betweenness centrality act as bridges or bottlenecks in the functional network; when links are interpreted as propagation instances, their disruption, for instance by increasing the resources available to that portion of the system, may result in widespread benefits.
• **Eigenvector Centrality.** This measure assigns a relative score to each node that is proportional to the score of its neighbours:
  (16) $$C_{E}\left(i\right)=\frac{1}{\lambda }\underset{j}{\sum }A_{ij}C_{E}\left(j\right),$$
  where λ is the largest eigenvalue of the adjacency matrix A. In other words, a node is as central as its neighbours are. The previous equation indicates that the centrality of nodes is given by the eigenvector of the largest eigenvalue of the adjacency matrix A, hence the name. In a delay network, nodes with high eigenvector centrality derive their importance from the fact of being connected with other central nodes, thus potentially forming a core.

#### 2.5.3 Isolated nodes analysis

In the case of delay functional networks, isolated nodes, i.e. nodes whose delays are not functionally dependent on other nodes, are a special and very interesting case. For instance, whenever such nodes exist, it may be relevant to understand which properties (in terms of e.g. availability of resources) is supporting their independence, for potentially replicating this across the network. This can be measured by a two-fold metric: the number of **Isolated Nodes (Inbound/Outbound)**:

(17) $$I_{in}=\overset{\left|V\right|}{\underset{i=1}{\sum }}1\left(\overset{\left|V\right|}{\underset{j=1}{\sum }}A_{ji}=0\right),$$

and

(18) $$I_{out}=\overset{\left|V\right|}{\underset{i=1}{\sum }}1\left(\overset{\left|V\right|}{\underset{j=1}{\sum }}A_{ij}=0\right).$$

In both cases, 1(⋅) is the indicator function that equals one when the condition is true, and zero otherwise.

#### 2.5.4 Temporal evolution of topological metrics

Transportation networks and their delay patterns can change over time due to seasonal variations in demand, changes in scheduling or operations, infrastructure developments, or external disruptions like weather events and industrial actions. It is therefore interesting not only to analyse general (or stable) patterns; but also how those evolve with time, to identify common trends and recurring structures. The previously described topological metrics can be calculated over any functional network; it is thus trivial to evaluate their evolution through time, by changing the corresponding time window of the data used in their reconstruction.

**Fig. 6:**
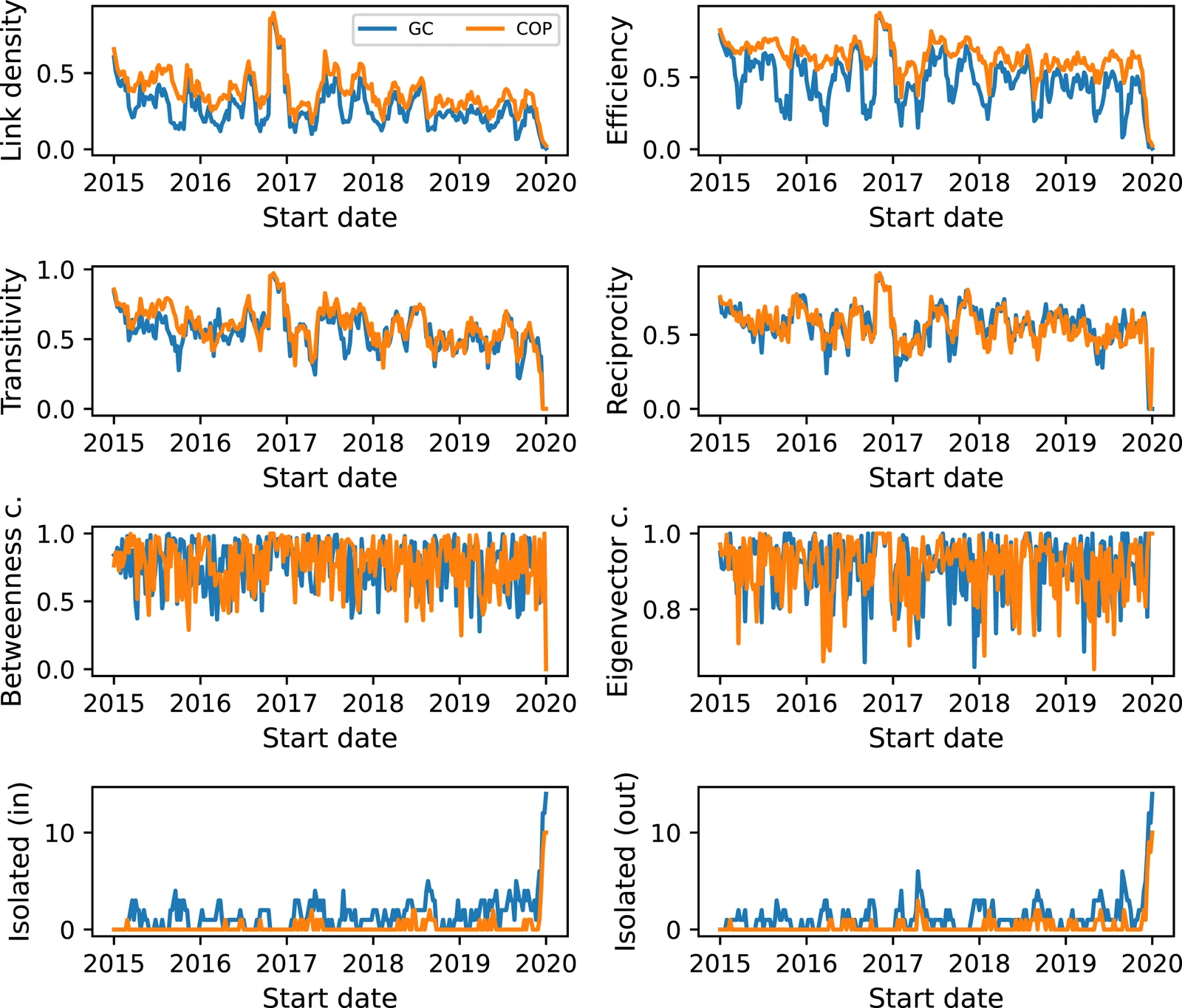
Example of the evolution of eight topological metrics described in Section 2.5, for the top-15 US airports, calculated using a rolling window of 60 days. Blue and orange lines respectively correspond to networks reconstructed using GC and COP.

To illustrate this point, Fig. 6 depicts the evolution over time of eight topological metrics previously described, for the data of the top-15 US airports from 2015 to 2019. The corresponding functional networks have been extracted using a rolling window of 60 days of length, shifted of one week for each point; time series have further been detrended using the Z-Score method with a periodicity of 24 h. Several interesting trends can be observed. As also obtained in Fig. 4, Fig. 5, the COP (orange lines) seems to detect more functional links than the GC (blue); the latter is also more sensitive to changes over time, with larger variations in metrics like the efficiency and number of isolated nodes.

#### 2.5.5 Normalisation of topological metrics

The analysis of the topological metrics of Fig. 6 has one important limitation: the values are not normalised according to the characteristics of the network under study. Let us explain this point by using the efficiency as an example. This topological metric strongly depends on the number of nodes and links in the network — as by definition denser networks will be able to propagate information in a more efficient way. Even assuming a constant number of nodes, this implies that the efficiency of two networks cannot directly be compared. To solve this, it is customary to use a normalisation procedure, in which the observed value of the topological metric is transformed using a Z-Score, calculated against the values of the same metric obtained in ensembles of random equivalent (i.e. same number of nodes and links) graphs (Zanin et al., 2018).

**Fig. 7:**
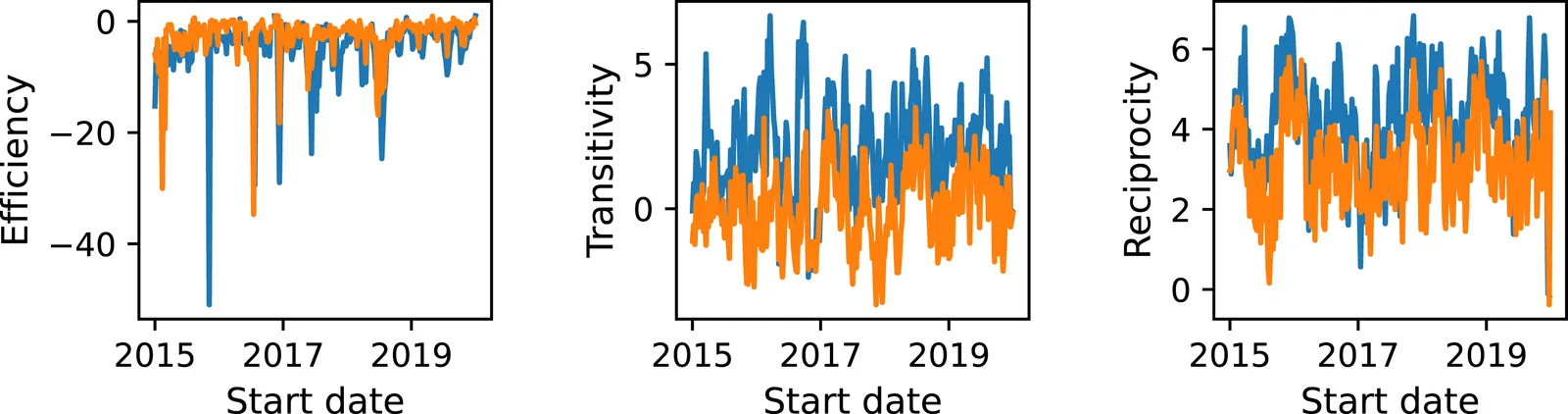
Temporal evolution of three metrics of Fig. 6, normalised using a Z-Score and ensembles of 1000 random equivalent networks. Blue and orange lines respectively correspond to networks reconstructed using GC and COP.

To illustrate this point, Fig. 7 depicts the evolution of three metrics previously reported in Fig. 6, this time normalised using a Z-Score. Let us consider the transitivity as an illustrative example. When considering the raw metrics, i.e. Fig. 6, the transitivity for GC and TE were essentially the same — average and standard deviation of respectively 0.554 ± 0.144 and 0.594 ± 0.143. On the other hand, the Z-Score normalised counterparts have substantially different means: 2.027 ± 1.711 for GC, and 0.009 ± 1.376 for TE. The metric normalisation can thus help in making explicit topological differences that arise when using multiple connectivity metrics.

In short, the practitioner reaching this stage has to take into account the kind of information that they want to obtain from the analysis. Whenever the aim is to simply describe the structure, e.g. to assess how many triangles are present, the raw (non-normalised) metrics are the correct option. On the other hand, if the goal is to compare networks, and furthermore understand if their structure is fundamentally different beyond what is consequence of a different link density, then the normalised version is a better solution.

## 3 The *delaynet* package: Implementation and usage

The *delaynet* package provides a complete implementation of the delay analysis framework described in Section 2, offering researchers and practitioners a powerful set of tools for analysing delay relationships in transportation networks. The package is available on PyPI and conda-forge, making it easily accessible to the research community. The package is developed following best coding practices, adhering to open-source principles, and includes a comprehensive Code of Conduct (https://github.com/cbueth/delaynet/blob/main/CODE_OF_CONDUCT.md) and Contributing Guidelines (https://github.com/cbueth/delaynet/blob/main/CONTRIBUTING.md), fostering a welcoming and collaborative community.

### 3.1 Package overview

The *delaynet* package is designed with a modular architecture that closely follows the framework illustrated in Fig. 1. This modular design allows users to select appropriate methods for each stage of the analysis process, tailoring the approach to their specific data characteristics and research objectives; and, whenever needed, use their own implementation of these methods. The package is organised into several core modules, each corresponding to a key component of the delay analysis framework:

• delaynet.preparation: Tools for data preparation and synthetic data generation.
• delaynet.detrending_methods: Implementation of various detrending techniques.
• delaynet.connectivities: Collection of connectivity measures for detecting relationships between time series.
• delaynet.network_reconstruction: Functions for reconstructing networks from time series data.
• delaynet.network_analysis: Tools for analysing and interpreting reconstructed networks.
• delaynet.utils: Utility functions supporting the core functionality.

The package can be imported, and its version checked using the code shown in listing 1.

**Figure:**
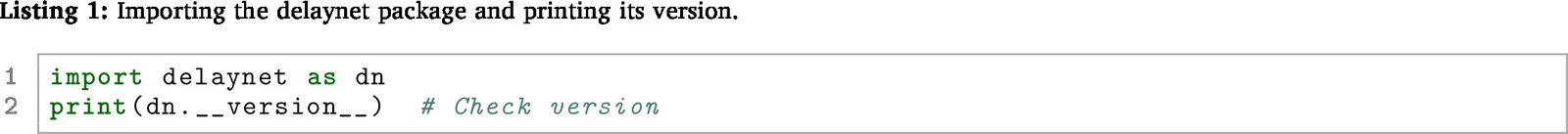

### 3.2 Data generation and preparation

The starting point of the analysis is a set of time series for each node in the network, which must be complete and of sufficient quality standards, as discussed in Section 2.1. For validation and testing of the analytical pipeline, the package supports synthetic data generation models, which are described below and are illustrated in listing 2.

Firstly, synthetic random time series with explicit causal relations between them can be generated through delayed causal networks. This method constructs networks through the systematic generation of adjacency matrices defining the network topology, weight matrices specifying connection strengths, and lag matrices determining the propagation of information. Data are then synthesised by drawing random values, and further adding delayed contributions from connected time series. The resulting time series exhibit controlled functional delay patterns where connections are activated probabilistically, simulating the sporadic nature of real transportation delay influences.

Secondly, the package incorporates the generation of transportation-specific synthetic time series through integration with the specialised simulation tool *SynthATDelays* (Büth and Zanin, 2025). Unlike comprehensive air transport simulators that aim for maximum realism at high computational cost, this method focuses on generating highly tuneable scenarios to test specific conditions and hypotheses. The *delaynet* package provides direct access to two predefined *SynthATDelays* scenarios:

• **Random connectivity scenario.** Simulates a set of airports randomly connected by independent flights with random and homogeneous en-route delays. This scenario allows customisation of the number of airports, number of aircraft, and buffer time between operations.
• **Independent operations with trends.** Creates two groups of two airports, in which flights connect airports within the same group but not across them. When trends are activated, delays are added at specific hours, generating spurious causality relations between airports despite no actual propagation pathways between groups.

**Figure:**
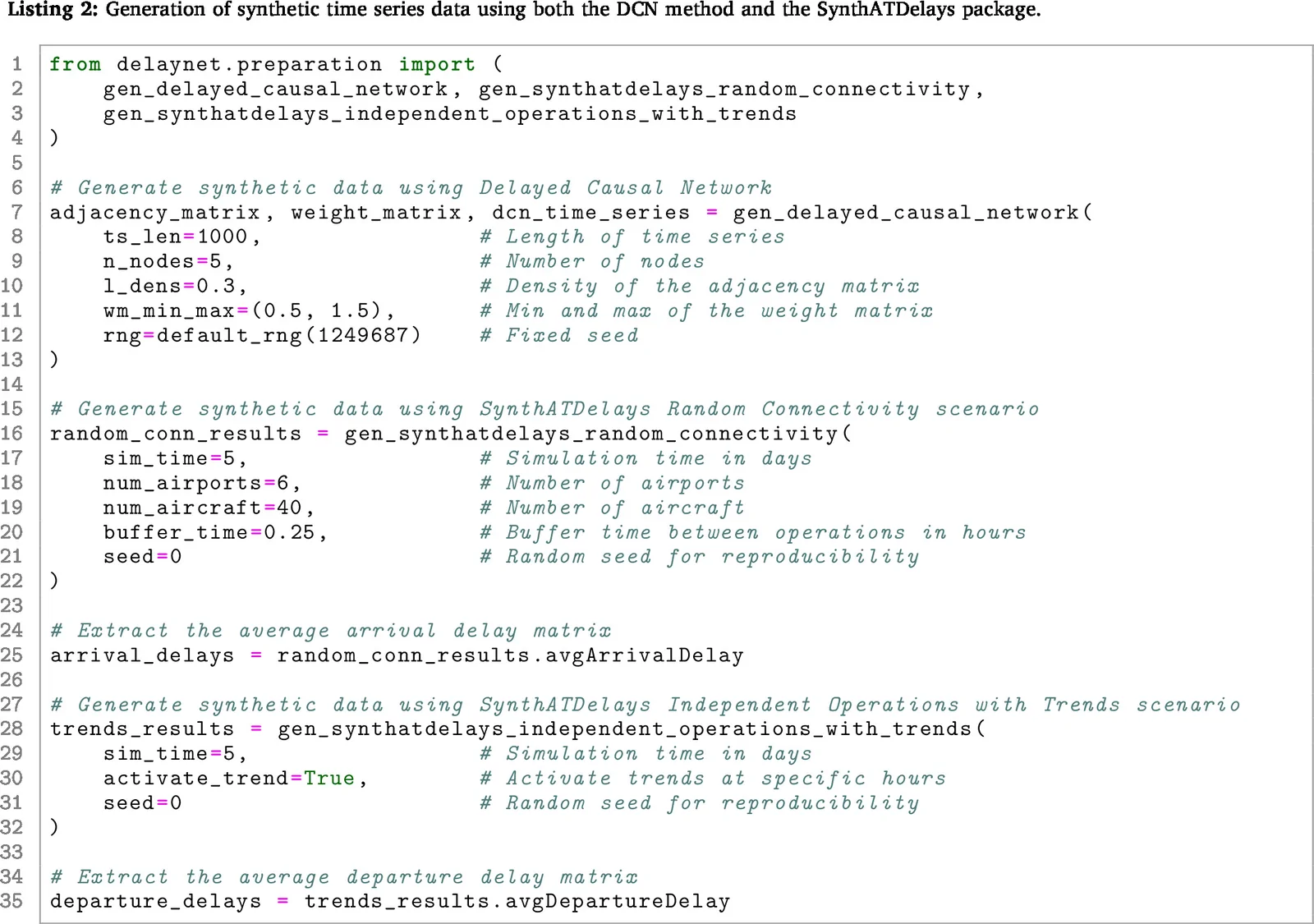

The functions used in listing 2 are:

• **Delayed Causal Network (DCN)**: The gen_delayed_causal_network function creates time series with explicit causal relationships and temporal delays between nodes, returning the adjacency matrix, weight matrix, and generated time series.
• **SynthATDelays**: Two specialised functions provide access to aviation-specific synthetic delay generation:
  – gen_synthatdelays_random_connectivity: Implements the Random Connectivity scenario with customisable parameters for airports, aircraft, and buffer times.
  – gen_synthatdelays_independent_operations_with_trends: Implements the Independent Operations with Trends scenario that can generate spurious causality relations.

These synthetic data generation capabilities enable researchers to validate their analytical pipelines against known ground truth, compare the performance of different connectivity measures under controlled conditions, and explore the impact of various parameters on delay propagation detection. The *SynthATDelays* integration is particularly valuable for transportation research, as it provides realistic delay patterns based on a simplified but accurate model of air transportation operations (Büth and Zanin, 2025).

### 3.3 Detrending algorithms

Time series detrending is an essential preparatory step that helps standardise data, remove trends, and make different time series comparable. The *delaynet* package provides a consistent interface for applying various detrending methods to time series data, including delta, second difference, and Z-Score detrending — see Section 2.2 for definitions. Examples of the corresponding code are reported in listing 3, and the list of available algorithms and parameters is presented in Table 1.

**Figure:**
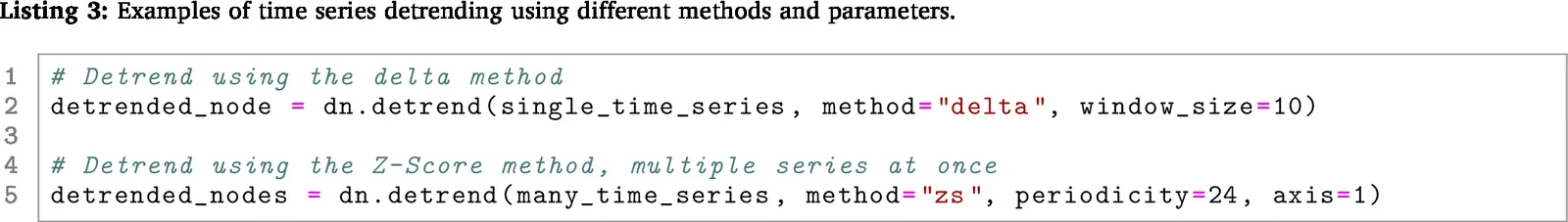

The unified interface allows users to easily experiment with different detrending methods and select the most appropriate one for the specific data characteristics and research questions. Switching detrending approaches only requires selecting the wanted method and passing its parameters, if needed. The input and output formats stay the same, meaning any following analysis — calculations and plotting — can be reused without modification.

**Table 1:** List of detrending algorithms included in *delaynet*. The second column reports the primary identifier to be used in the *method* parameter of the *dn.detrend* function, while the third column lists all additional parameters that can be defined. Additional method aliases are supported; see detrending documentation for the complete identifier list.

Algorithm	Identifier	Parameters
Identity	identity	–

Delta	delta	*window_size*: window size to use for calculating the mean.

Second Difference	2dt	–

Z-Score	zs	*periodicity*: expected periodicity of the time series, i.e. reoccurrence of the same pattern.
		*max_periods*: maximum number of periods to consider before and after the current value.

### 3.4 Connectivity analysis

After detrending, the next step is to quantify the relationships between delay time series of different components of the transportation network. The *delaynet* package implements various connectivity measures that can detect and measure how delays in one part of the network influence delays in other parts, accounting for the time it takes for these influences to propagate.

**Figure:**
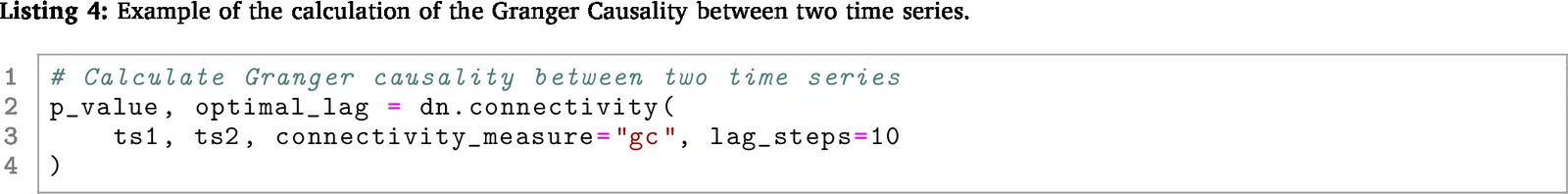

The package provides a simple interface for applying connectivity measures, as illustrated in listing 4. The implemented measures include Linear Correlation (LC), Rank Correlation (RC), Granger Causality (GC), Mutual Information (MI), Transfer Entropy (TE), and Continuous Ordinal Patterns (COP) — see Section 2.3 for definitions and details. Note that each measure has some specific parameters that can be defined by the user, and which are listed in Table 2.

For each connectivity measure, the package determines the optimal lag by systematically testing different time lags and selecting the one that produces the strongest relationship, which is always determined by the lowest p-value. This process is handled by the find_optimal_lag utility function, which ensures consistent behaviour across all connectivity measures.

**Table 2:** List of connectivity measures included in *delaynet*. The second column reports the primary identifier to be used in the *connectivity_measure* parameter of the *dn.connectivity* function, while the third column lists all additional parameters that can be defined. Additional method aliases are supported; see measure documentation for the complete identifier list.

Measure name	Identifier	Parameters
Linear correlation	lc	*lag_steps*: time lags to consider.

Rank correlation	rc	*lag_steps*: time lags to consider.

Granger Causality	gc	*lag_steps*: time lags to consider.

Mutual Information	mi	*lag_steps*: time lags to consider.
		*approach*: approach to use.
		*hypothesis_type*: type of hypothesis test to use, either “permutation_test” or “bootstrap”.
		*n_tests*: number of iterations or resamples to perform within the hypothesis test.
		**mi_kwargs: additional estimator parameters.

Transfer Entropy	te	*lag_steps*: time lags to consider.
		*approach*: approach to use.
		*hypothesis_type*: type of hypothesis test to use, either “permutation_test” or “bootstrap”.
		*n_tests*: number of iterations or resamples to perform within the hypothesis test.
		**te_kwargs: additional estimator parameters.

Continuous Ordinal Patterns	cop	*lag_steps*: time lags to consider.
		*p_size*: size of the ordinal pattern.
		*num_rnd_patterns*: number of random patterns to consider.
		*linear*: start with the identity pattern.

### 3.5 Network reconstruction

The network reconstruction involves applying connectivity measures to each pair of time series in the dataset, resulting in a matrix of p-values and a matrix of optimal lags. The *delaynet* package provides the reconstruct_network function to streamline this process, as demonstrated in listing 5.

**Figure:**
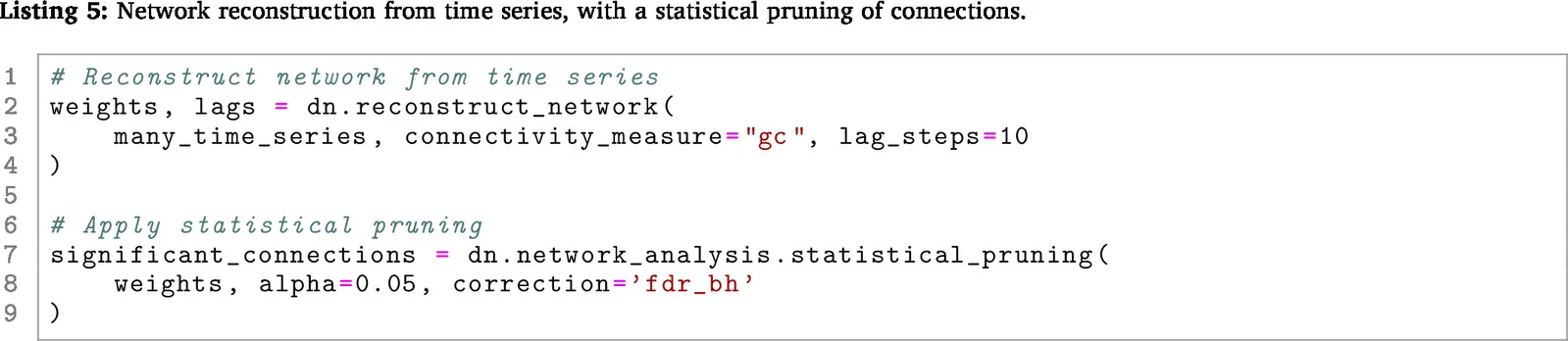

Note that the reconstruct_network function internally includes three steps, namely: the pairwise analysis, which, for each node pair (i,j), computes the p-values $p\left(X_{i},X_{j},\tau \right)$ for the connectivity measure across lags $\tau =1,\ldots ,\tau_{\max}$ or a specified list of lags; the lag optimisation, which selects the lag yielding the most significant connection $\tau_{ij}^{*}=\arg\min_{\tau }p\left(X_{i},X_{j},\tau \right)$; and finally the matrix reconstruction, which yields the weight matrix $W_{ij}=p\left(X_{i},X_{j},\tau_{ij}^{*}\right)$ and the lag matrix $L_{ij}=\tau_{ij}^{*}$.

After the reconstruction, the package provides several thresholding strategies to convert the continuous p-value matrix into a binary adjacency matrix. These include a statistical-thresholding, which retains connections with p-values below a specified significance level; and a False Discovery Rate control, which adjusts the threshold to control the expected proportion of false positives among all detected connections, with support for various correction methods from statsmodels.stats.multitest.multipletests (Seabold and Perktold, 2010).

### 3.6 Network analysis

Once the delay functional network has been reconstructed, the *delaynet* package provides a comprehensive set of network analysis tools to extract meaningful insights about the structure and dynamics of delays, as shown in listing 6.

**Figure:**
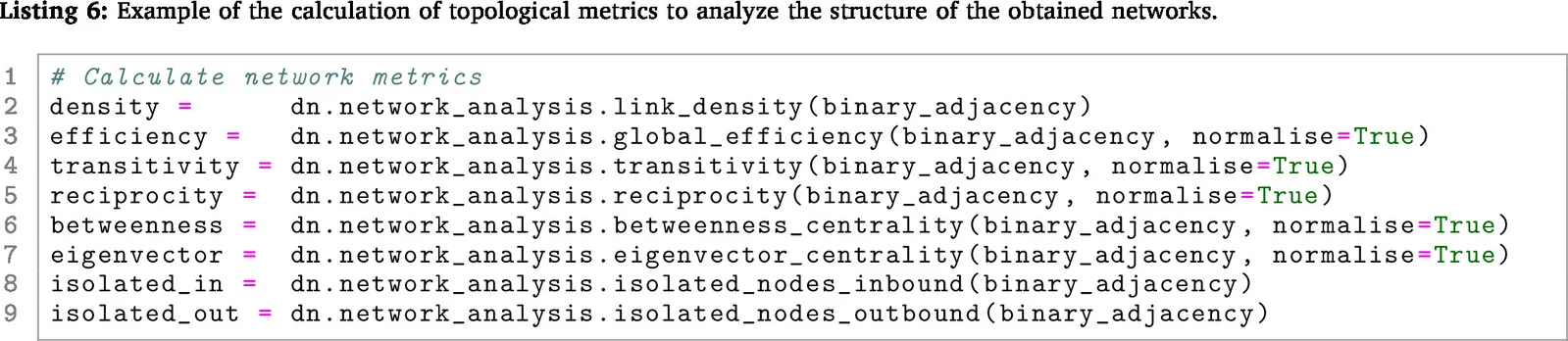

The full list of network topological metrics, which have been selected for their relevance in transportation delay networks, includes: link density, global efficiency, transitivity, reciprocity, betweenness centrality, eigenvector centrality, and number of isolated nodes. Beyond these, the output matrices W and L can be analysed by any other network analysis tool, providing flexibility for researchers with specific analytical needs. Additionally, each topological metric can be called with a parameter *normalise*, which normalises the result according to what observed in an ensemble of random equivalent networks — see Section 2.5.5.

### 3.7 Package availability and documentation

The *delaynet* package is readily available through standard Python package repositories and can be installed using the commands shown in listing 7.

**Figure:**
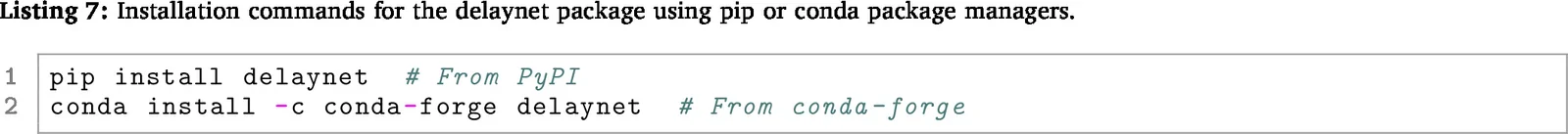

The documentation is available online, see https://delaynet.readthedocs.io, and includes up-to-date references and examples. The documentation demonstrates the complete workflow from data preparation to network analysis, providing researchers with a solid foundation for applying the package to their own transportation delay data. The package is versioned with Zenodo, ensuring reproducibility and proper citation in scientific research. Finally, *delaynet* is continuously tested for Python ≥  3.10. For the latest updates and detailed usage instructions, users are encouraged to visit the package documentation.

## 4 Case study: Swiss long-distance rail delays (2022–2025)

We illustrate the *delaynet* workflow on Swiss Federal Railways (SBB) realised timetable data (*istdaten*) accessed via https://opentransportdata.swiss/. The feed provides, for each stop event, scheduled and realised arrival/departure times with reliability statuses. We exclude cancelled services (FAELLT_AUS_TF) and pass-throughs (DURCHFAHRT_TF) and prioritise REAL over PROGNOSE and GESCHÄTZT (i.e. predicted) when deriving executed times. Delays are computed as realised minus planned times in seconds. We consider data from January 2022 to August 2025, and focus on long-distance SBB-operated services, specifically line prefixes IC, IR, EC, ICE, and TGV, operated by SBB (BETREIBER_ABK = ’SBB’) with PRODUKT_ID = Zug. Single-month views presented below refer to August 2025.

From these events we construct station-level arrival-delay time series in fixed 15-minute bins. The 15-minute resolution balances temporal detail against data sparsity: it is of the same order as passenger transfer intervals at major hubs, while retaining more observations per bin than a finer resolution. Five-minute bins are more susceptible to sparsity during low-traffic periods, whereas 30- and 60-minute bins may smooth short-timescale delay dependencies. Appendix C shows that the qualitative evolution of the network characteristics considered here is consistent across these resolutions. For each station and bin we aggregate the delay by summation, yielding a delay matrix $D_{t,s}$ with columns ordered as the top-50 busiest long-distance stations by event volume in July 2025. To reduce the non-stationarity due to daily cycles, we apply the Z-Score detrending with daily periodicity, i.e. periodicity of 96 for 15-minute bins; for a full discussion about the non-stationarity of these time series, and the rationale for using a daily detrending period, see App. A.

We reconstruct a directed functional network of delay associations using *delaynet*’s unified interface. For every ordered station pair we evaluate the Granger Causality across a set of candidate lags {1,2,…,16,18,20,24,28,32} (time steps of 15 min, i.e., up to eight hours with intermediate values), and retain the minimum p-value together with its optimal lag. This ensures that the best lag is retained for each pair of stations, thus accounting for the presence of different relationships between time series. Note that increasing this maximum lag has a minimal impact on the results, as discussed in App. B. The resulting p-value matrix is statistically pruned at significance level α=0.01 using Benjamini–Hochberg FDR control. We then compute standard metrics (link density, reciprocity, global efficiency, transitivity) and node centralities (eigenvector, betweenness; all reported as normalised scores in the figures). Community structure is obtained with the Louvain algorithm applied to the undirected (symmetrised) projection of the pruned network. Using 15-minute arrival-delay time series, the August 2025 functional network exhibits a link density of 0.5143; there are 0 nodes without incoming connections and 0 nodes without outgoing connections; the transitivity equals −1.8201 and the reciprocity equals 12.2859. Note that both the transitivity and the reciprocity are here reported as Z-Scores relative to a random null model, and not as raw ratios (see Section 2.5.5). A high reciprocity Z-Score should be interpreted with care: it only indicates that the number of bidirectional connections is significantly higher than expected in the random baseline network (here, about 12 standard deviations above the ensemble mean), but does not imply that the vast majority of connections in the network are bidirectional. Note further that this significance is much lower than what observed in the underlying physical network, see App. D. Geographic maps are rendered in the Swiss LV95 projection (EPSG:2056) with an OpenRailwayMap overlay for context. In terms of the *delaynet* API, this corresponds to the concise sequence detrend → reconstruct_network → network_analysis.statistical_pruning→ network_analysis metrics (link density, reciprocity, global efficiency, transitivity, eigenvector, and betweenness centralities).

**Fig. 8:**
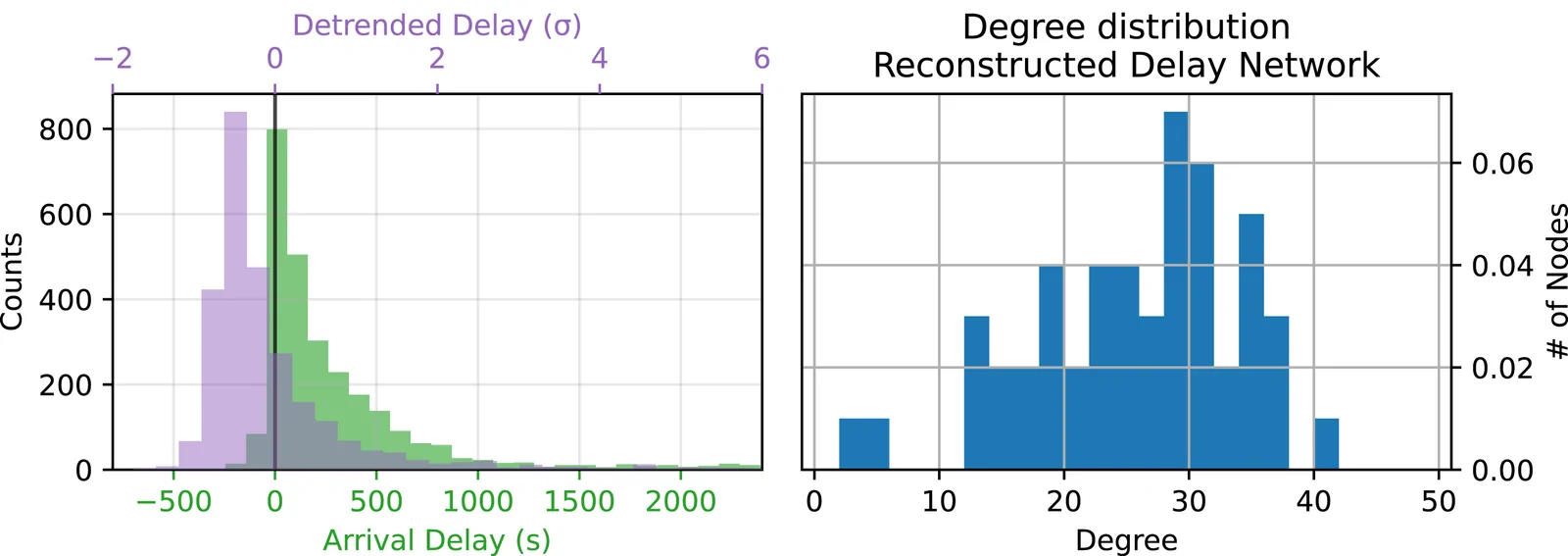
Analysis of delay data. (Left) Distribution of arrival delays at Zürich HB in August 2025 (15-minute bins). Green and magenta bars respectively correspond to raw (bottom X axis) and detrended (top X axis) delays. (Right) Out-degree distribution of the reconstructed arrival-delay functional network for August 2025 across the top-50 stations, using bars centred between left-inclusive bin edges.

The left panel of Fig. 8 presents, for August 2025, the empirical delay distribution at Zürich HB, while the right panel depicts the out-degree distribution of the reconstructed arrival-delay network across the top-50 stations. The former summarises local variability in operational performance, while the latter characterises the heterogeneity of inferred functional associations by node out-degree. For Zürich HB, the arrival-delay distribution is markedly heavy-tailed with strong positive skewness (4.886) and leptokurtosis (excess kurtosis, Fisher of 39.282); after the daily Z-Score detrending, both the skewness and the excess kurtosis increase to respectively 5.947 and 52.106. Across the most skew stations, tail-heaviness varies considerably, e.g.: Basel SBB skewness =7.325, excess kurtosis =92.465; Lausanne skewness =5.950, excess kurtosis =64.637; Olten skewness =4.990, excess kurtosis =44.589; Genève skewness =16.514, excess kurtosis =424.648. This indicates that, globally, the majority of trains arrive on time or with small delays, while rare but severe incidents (e.g. accidents, adverse weather, technical failures) generate the pronounced right tails. Genève stands out as particularly volatile (skewness ≈16.5, excess kurtosis ≈425), indicating a higher propensity for extreme outliers than at other major hubs. Notably, detrending increases both skewness and kurtosis at Zürich HB, suggesting that once daily cycles are removed, the residual dynamics are even more dominated by rare, high-impact events.

**Fig. 9:**
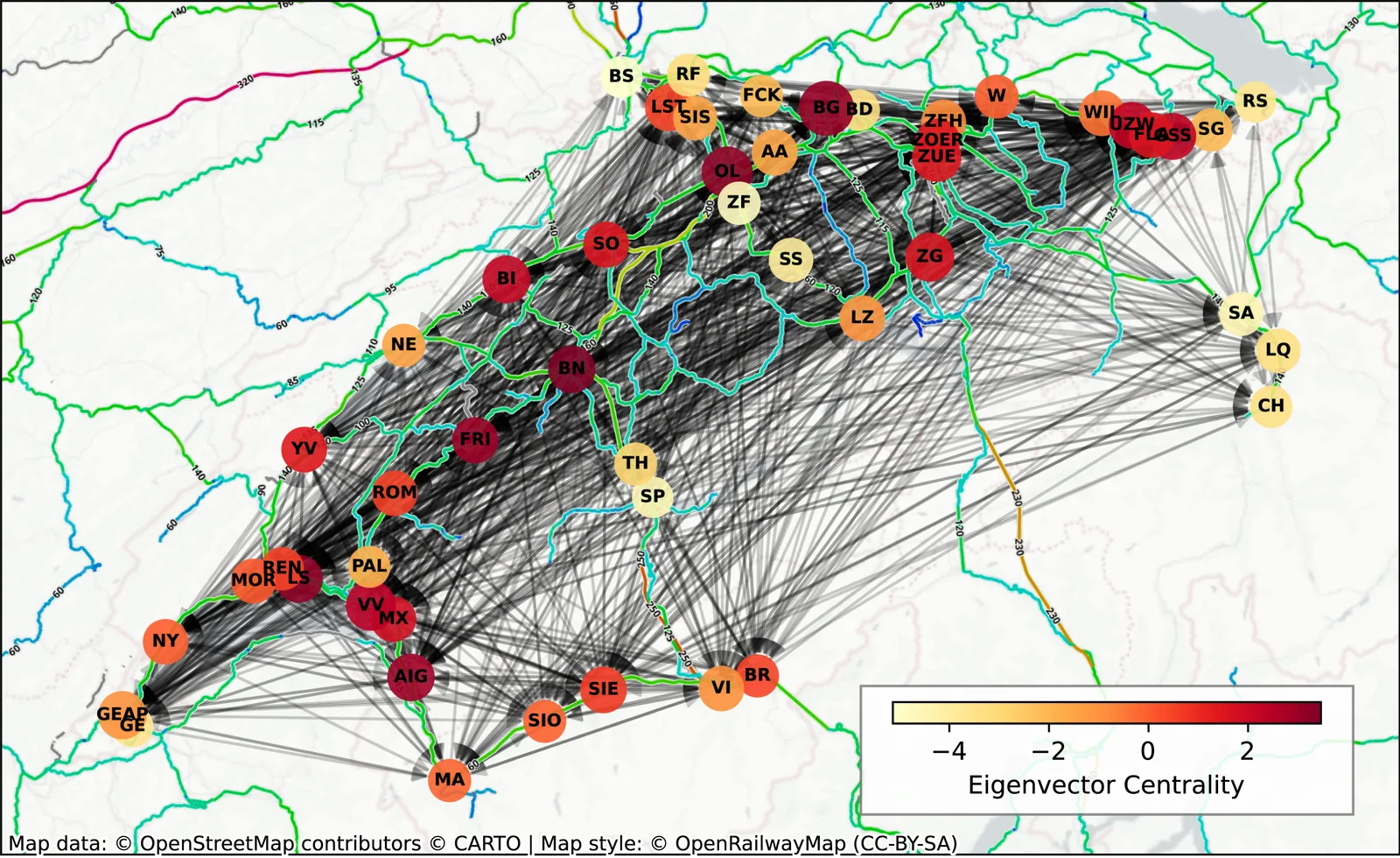
Pruned arrival-delay functional network for August 2025. Node colour encodes eigenvector centrality (normalised), while node size encodes betweenness centrality (normalised). Map in LV95 (EPSG:2056) with OpenRailwayMap overlay, with colour representing the maximum speed on the rail segment. Basemap data courtesy of OpenStreetMap contributors, CARTO, and OpenRailwayMap (CC-BY-SA). See Table 3 for station names and exact centrality values.

Fig. 9 displays the pruned arrival-delay network on the map, with eigenvector centrality encoded by node colour and betweenness centrality by node size. Eigenvector centrality highlights stations embedded in globally central structures, whereas betweenness emphasises bridge nodes that represent key points of functional association. In August 2025, betweenness is dominated by junctions in the Aargau/Mittelland such as Brugg AG and Olten, with notable bridge roles also at Vevey and Zürich HB; by contrast, eigenvector centrality peaks at Bern and Lausanne, with Olten and Brugg AG also ranking highly. For station abbreviations, full names, community membership, and exact centrality values, see Table 3.

**Fig. 10:**
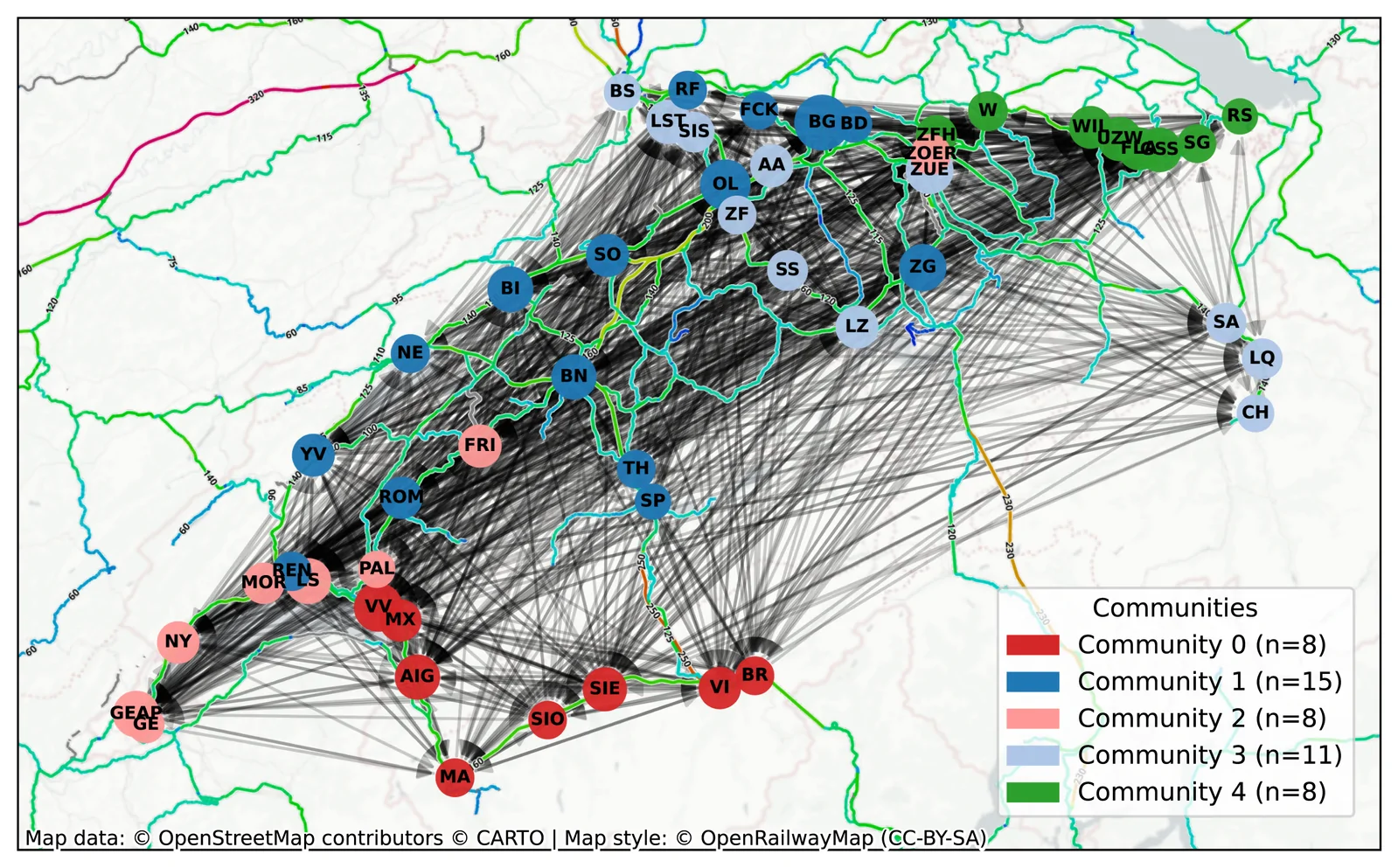
Louvain communities of the August 2025 network, computed on the symmetrised adjacency with undirected weights equal to the number of directed links (0/1/2). Communities reflect corridor-like structures (abbreviations shown). Projection and basemap as in Fig. 9. Station names provided in Table 3.

Fig. 10 further shows the community partition of the same network using the Louvain algorithm, computed on the symmetrised graph where the undirected edge weight equals the number of directed links between a pair (0, 1, or 2). The August 2025 partition yields five communities that align with corridor-like structures; see Table 3 for the station composition (abbreviations and full names) sorted by betweenness within each community. These clusters seem to indicate that delays are preferentially associated along main operating corridors; at the same time, the presence of nodes like ZOER (Zürich Oerlikon) in the Lémanic–Fribourg community highlights non-local ties created by through-services and long-range dependencies. The practitioner should nevertheless exercise caution when interpreting these results. It is known that stochastic modularity maximisation algorithms, like the Louvain one, can yield inconsistent results, which are further dependent on the FDR pruning procedure — see App. C for a detailed discussion. A full evaluation of the community structure would therefore require the comparison of the solution yielded by multiple algorithms, to achieve a consensus viewpoint; and eventually the integration of external information about operations.

**Table 3:**
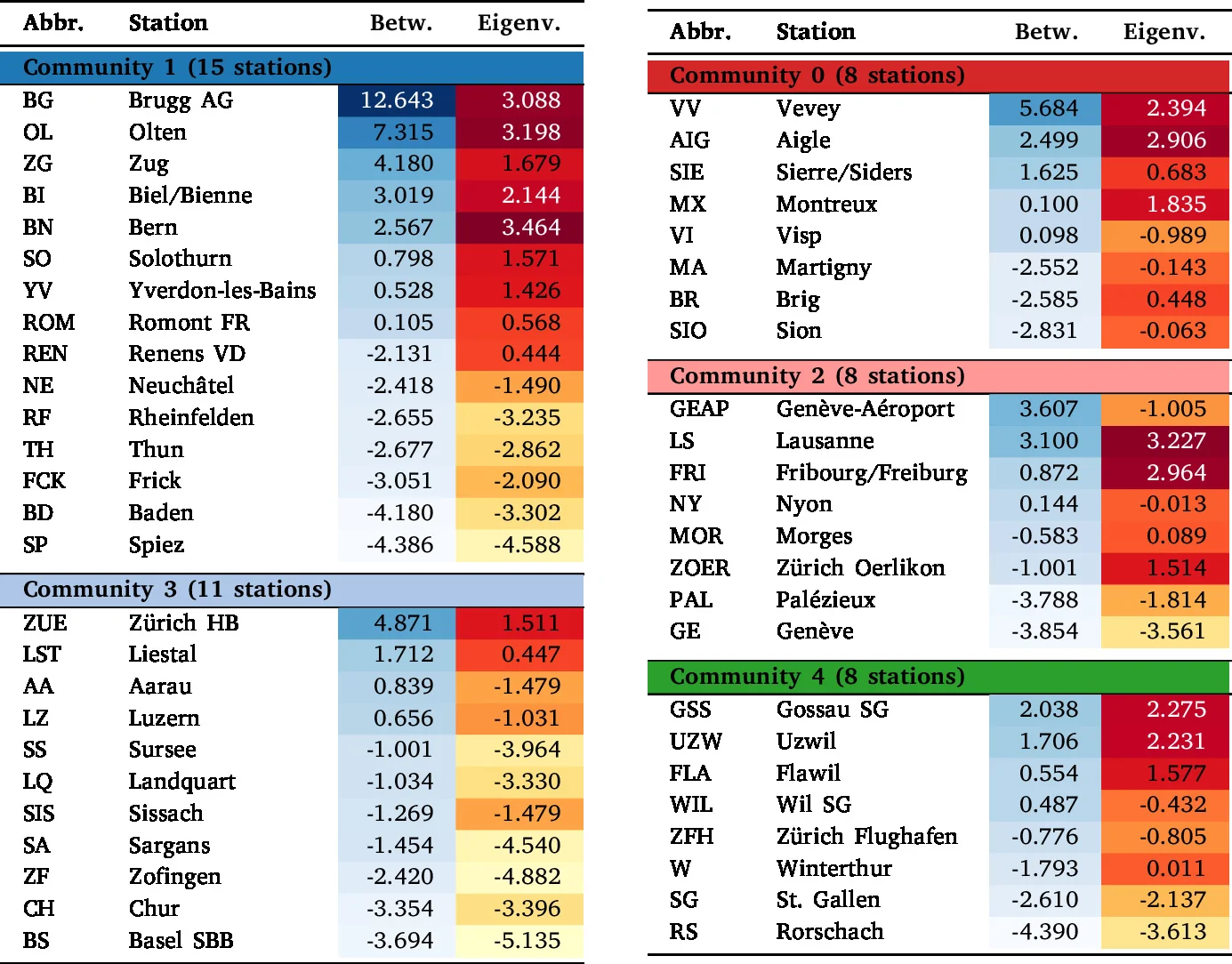
Network centrality measures by community in the Swiss railway delay network (August 2025). Communities are identified using the Louvain algorithm on the symmetrised adjacency matrix. Station abbreviations correspond to those shown in Figs. 9 and 10; full station names are provided for reference. Centrality values correspond to normalised scores, with colour coding indicating relative magnitudes within each measure.

**Fig. 11:**
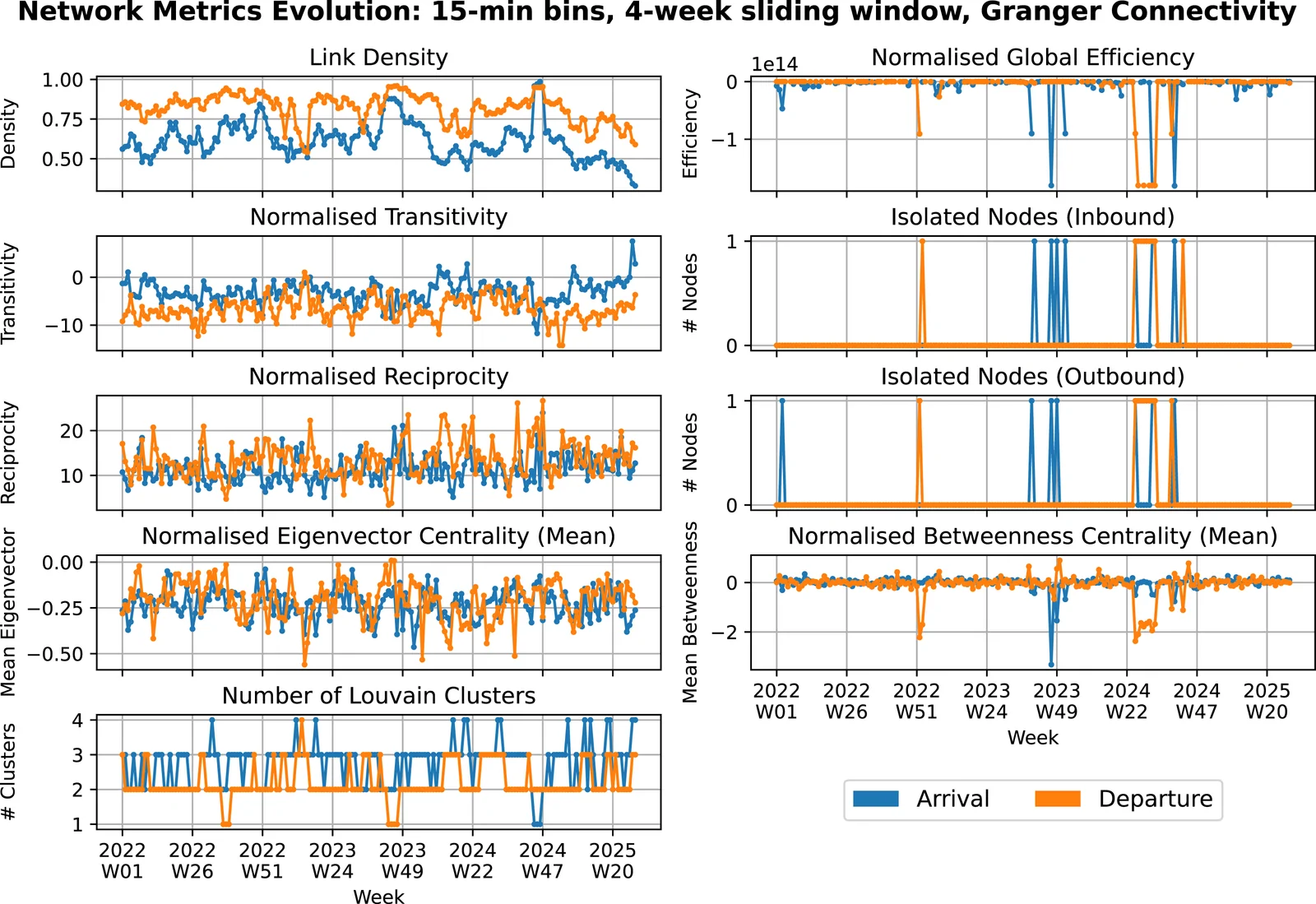
Evolution of topological metrics for 15-minute arrival (blue) and departure (orange) delays in the Swiss rail system, calculated using a 28-day sliding-window. The normalised metrics, including the reciprocity and the global efficiency, are reported as Z-Scores relative to a random null model, and not as raw ratios (see Section 2.5.5). See panel titles for metric names.

Finally, Fig. 11 depicts the evolution of some topological metrics through time. These have been estimated using a rolling-window approach: networks are reconstructed on 28-day windows sliding weekly (Monday–Sunday), for then calculating the corresponding metrics — see also App. C for an analysis of the sensitivity to the sliding window size. While prima facie the depicted evolutions seem random, some patterns emerge. To illustrate, the link density and the reciprocity for the arrival delay functional networks present spikes during the last weeks of each year; while, on the other hand, the transitivity drops during those same dates. Note that the transitivity and the reciprocity here reported correspond to the normalised versions of both metrics (see Section 2.5.5), hence their variations are not a direct consequence of the change in the number of links. On the contrary, it may be concluded that these changes are the result of an increase in traffic during the holiday season; the higher level of stress imposed on the available resources implies that delays become more interconnected, resulting in a denser, yet less structured, functional network. Still, as in the previous case, the complete interpretation of these results would require operational information beyond what here presented. It is finally worth noting some abnormal values, specifically for the Z-Score of the Global Efficiency (see top right panel of Fig. 11). These are due to the high link density of the functional network, which results in a limited variability in the metric value for the random ensemble, and therefore in extremely large (in absolute terms) Z-Scores. Beyond the specific values, these have to be interpreted as instances of networks whose structure is clearly not compatible with the null model.

## 5 Discussion and conclusions

In this contribution we reviewed the main elements and challenges of the use of functional network representations to study delay relationships in transportation systems. In spite of many important results that have been obtained by the research community using this approach, the interested practitioner has also to face a large number of options, e.g. in terms of how to preprocess the data, which connectivity metric to apply, or how to evaluate the resulting structures. We here discussed these options, reviewing existing alternatives, highlighting limitations, and showing their impact in the final results through a series of examples drawn from air transport.

As already discussed in the introduction, the reconstruction of functional networks should not be understood as the complete solution to the analysis of transportation systems, but rather as a tool complementary to other approaches. More in detail, functional representations are helpful in describing the interactions between the elements of the system, but they are also hindered by three limitations. Firstly, relationships mostly describe correlations; even when using causality metrics, as the Granger Causality test, these may not correspond to genuine causality due to the presence of confounding effects. Secondly, while functional networks allow one to establish that a relationship between the delays at two stations exists, they do not explain the underlying reason - e.g. which elements or policies are responsible for such relation. Thirdly, they do not allow to simulate what-if scenarios, and thus cannot directly guide interventions in the system. These limitations notwithstanding, functional networks can be used as a first step in a more complete analysis, to identify relevant elements and relations to be later studied using, e.g., micro-scale simulations.

We complemented this theoretical discussion with *delaynet*, a holistic framework for analysing delay relationships in transportation networks. It integrates time series analysis techniques, network reconstruction methodologies and complex network metrics to enable a nuanced understanding of the structures created by (and supporting) the potential propagation of delays. The *delaynet* framework consists of five key components that together form a systematic approach: data preparation, detrending methods, connectivity analysis, network reconstruction, and network analysis. Each of these components is designed to address a specific aspect of delay analysis. By providing these in a unified package, it simplifies the analysis of real-world data and ensures that no essential steps are skipped, which in turn especially benefits practitioners not familiar with these techniques. At the same time, the package is also highly flexible; this allows for the application to different transportation modes and the accommodation of various data types and time scales, but also the integration within larger data analysis pipelines. The methods employed are founded upon well-established theories from statistical physics, information theory and network science; and, whenever available, leverage existing open-source libraries to foster validation and robustness.

The use of *delaynet* has been illustrated through an analysis of Swiss long-distance rail operations between 2022 and 2025. Note that, while functional network analyses have frequently been performed on air transport data (Zanin, 2015, Zanin et al., 2017, Pastorino and Zanin, 2021, Jia et al., 2022, Wang et al., 2022, Feng et al., 2024, Gil-Rodrigo and Zanin, 2024), to the best of our knowledge this is the first instance of such type of study on rail data. We have shown how detrended station-level delay series, lag-aware connectivity, and statistically-pruned networks yield interpretable spatial structures and temporal dynamics — see Section 4. These structures are further different from the topology of the underlying physical network, i.e. of how stations are physically connected by trains visiting them in a sequential fashion — see App. D for full results. While the physical backbone is mainly static, the functional network’s evolution (see Fig. 11) highlights time-local dependencies that deviate from the underlying infrastructure. In addition, the stations most central in both representations are highly different (see Fig. 20). This application underscores both the practical usefulness of the pipeline and avenues for further refinement (e.g., alternative estimators, robustness checks, and operational validation).

Notwithstanding the advantages of the *delaynet* framework, it is important to acknowledge its limitations and associated challenges. First of all, the framework requires high-quality time series data with sufficient temporal resolution and coverage. While not being something specific of the proposed package, the practitioner has to be aware of the data limitations, as well as of their specific properties — to illustrate, air transport delays can be calculated against originally filed or last filed flight plans, or using gate arrival times as opposed to touch-down times. Secondly, in spite of several optimisation strategies, some methods can be computationally intensive for large transportation networks, i.e. for a large number of nodes; but also when using sophisticated connectivity measures, or multiple parameters have to be compared. Thirdly, most methods included in the framework involve several parameters that need to be selected appropriately, which may require domain expertise or extensive experimentation to optimise for specific transportation systems. This also intermingles with the problem of interpreting the results; to illustrate, inasmuch as different connectivity measures describe different aspects of the propagation of delays, results yielded by them have to be carefully interpreted and compared. In synthesis, it would be ill-advised to apply *delaynet* as a black-box. To transition from such descriptive monitoring to a diagnostic understanding, it is necessary to resort to external event logs (e.g., weather data, infrastructure failures, or specific holiday schedules). Linking the detected topological fluctuations to these real-world events is a crucial step in moving from ‘when‘ and ‘where‘ a structural change occurred to ‘why‘ it happened.

As a final limitation, while *delaynet* includes all fundamental steps and analysis tools, we also acknowledge that many more have been proposed in the literature — as illustrated by the huge variety of topological metrics that have been published in different contexts (Costa et al., 2007). It is our intention to keep evolving the package, also by accepting suggestions and comments from users. Therefore, the interested reader should also check the online documentation at https://delaynet.readthedocs.io for the most up-to-date list of available options.

## CRediT authorship contribution statement

**Carlson Moses Büth:** Writing – review & editing, Writing – original draft, Visualization, Validation, Software, Methodology, Investigation, Formal analysis, Data curation, Conceptualization. **Massimiliano Zanin:** Writing – review & editing, Writing – original draft, Validation, Supervision, Project administration, Methodology, Investigation, Funding acquisition, Conceptualization.

## Declaration of competing interest

The authors declare that they have no known competing financial interests or personal relationships that could have appeared to influence the work reported in this paper.

## Data Availability

**Accession codes**: The *delaynet* package (version 0.3.3) is archived on Zenodo, at https://doi.org/10.5281/zenodo.16875272. **Documentation**: The full documentation is available at https://delaynet.readthedocs.io/. **Source Code**: The source code is publicly available on GitHub: https://github.com/cbueth/delaynet.

## Appendices. Supplementary data

MMC S1 Appendices A–D: stationarity and detrending, lag sensitivity, topology stability, and physical-network comparison. MMC S1
