# Supplementary material for: Functional connectivity networks for transportation delay analysis: From theory to software
Source: Transp Res Interdiscip Perspect. 2026 Sep;39:None. doi: 10.1016/j.trip.2026.102187 (PMC13569785; doi:10.1016/j.trip.2026.102187)
Supplement: Supplementary file 1 [file mmc1.pdf]

A. Stationarity and detrending

As discussed in Sec. 2.2, an aspect of the data that can lead to false results, and especially spurious causality relationships, is their non-stationarity. A Z-Score detrending process was therefore applied in Sec. 4 when analysing delay time series of Swiss trains; we here further detail what type of non-stationarity was initially present, and the result of applying such process.

Several statistical tests are available in the literature to check for the presence of non-stationarities in time series; we here specifically consider two of the most well-known ones, i.e. the Augmented Dickey-Fuller (ADF) unit root test and the Kwiatkowski-Phillips-Schmidt-Shin (KPSS) test for stationarity. It is important to note that they test opposite hypotheses: the former tests for the null hypothesis that there is a unit root, hence a non-stationarity, while the latter that the time series is stationary. Fig. 12 reports the distribution of the p-values obtained by both tests on the raw (i.e. not detrended) time series, see the blue bars. Notably, both tests agree in detecting no instances of non-stationary time series; it may prima facie be concluded that the detrending process is not needed in this instance.

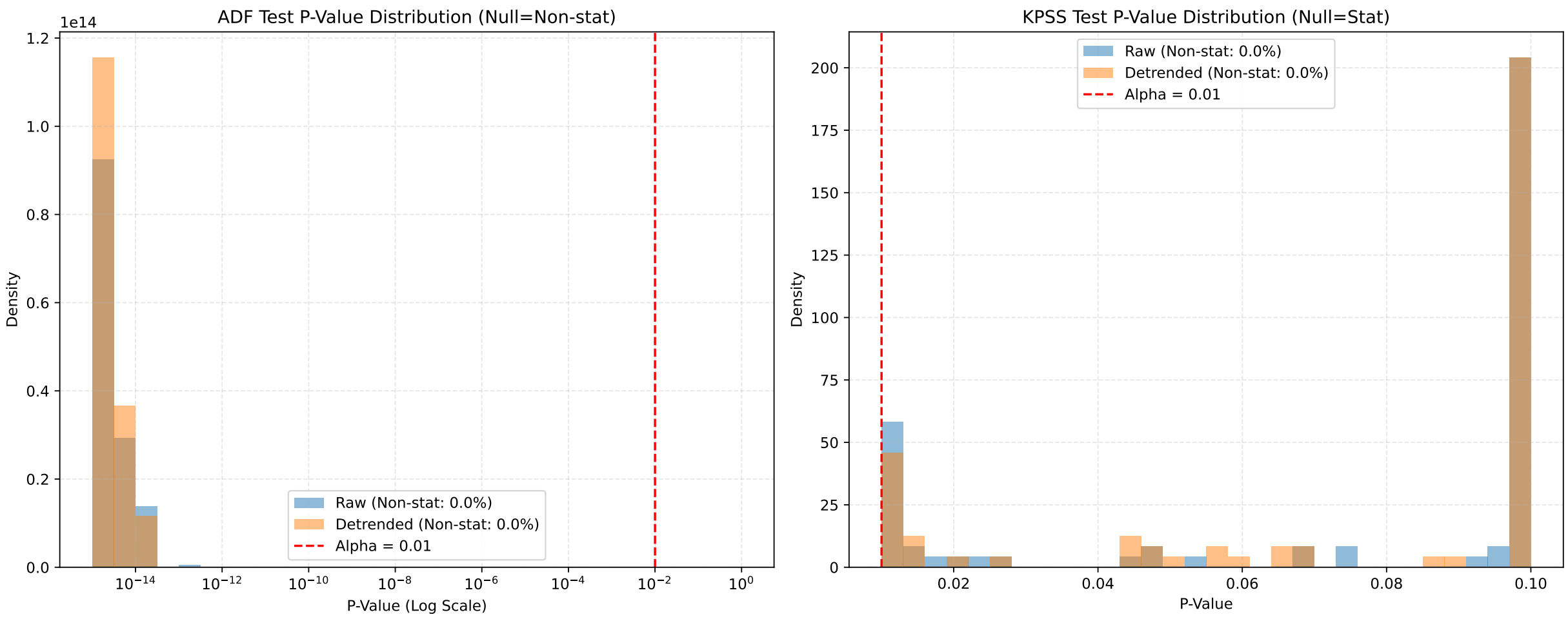

Figure 12: Stationarity of SBB delay time series. Left and right panels report the distribution of p-values yielded by respectively the ADF and KPSS tests, for the raw (blue bars) and detrended (orange bars) time series. The vertical red dashed lines indicate a statistical threshold $\alpha = 0.01$.

In order to confirm this, Fig. 13 reports the average correlogram of all raw time series, i.e. the correlation of the time series with themselves over different time lags - see blue line. Important peaks can be appreciated, with the most prominent one corresponding to 24 hours. In other words, and in spite of what reported by the previous tests, the time series are not stationary, and recurrent delay patterns appear with a periodicity of one day. These results both support the use of a detrending process, and further suggest that the detrending period of 24 hours should be considered. After the detrending process has been performed, results from the stationarity tests are mostly unchanged (orange bars in Fig. 12); and the periodic patterns in the correlogram are further strongly reduced (orange line in Fig. 13).

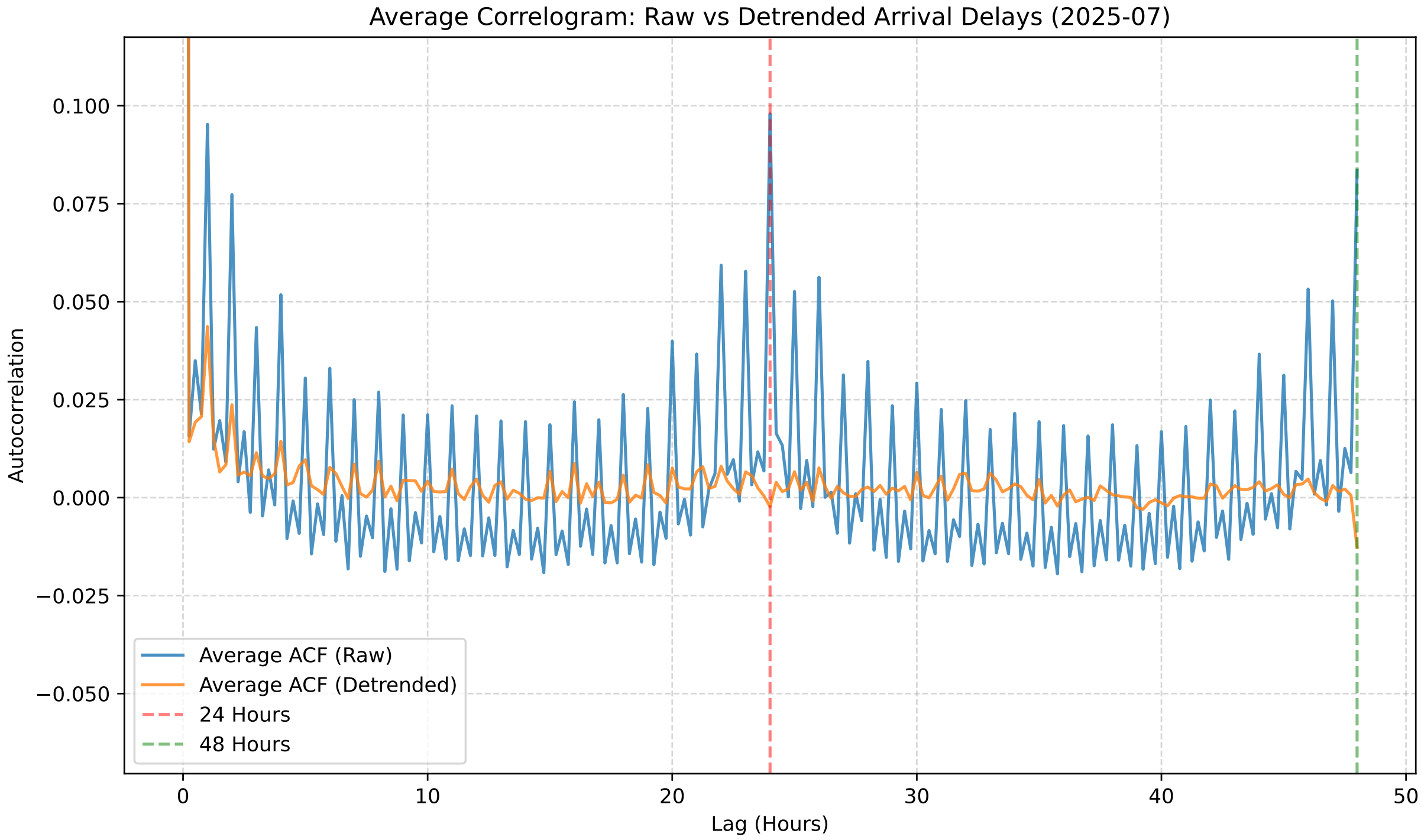

Figure 13: Average correlograms of the raw (blue line) and detrended (orange line) SBB delay time series, as a function of the lag in hours. Vertical dashed lines indicate the one day and two days periodicities.

B. Time lag structure

As described in the main text, the choice of the maximum lag in the Granger Causality can potentially have major effects on the obtained results. In the event that the true time required to describe the relationships is not known, this can be seen as a balance between shorter and longer lags: the former option ensures a higher discriminative power, but the latter avoids the problem of potentially missing long relationships.

As an example of this balance, we have performed a sensitivity analysis on the maximum lag parameter (max_lag) for the Granger Causality test using the 80 most frequented stations in the SBB network. The results, depicted in Fig. 14, demonstrate that the number of statistically significant links increases rapidly for small lags, but begins to saturate as the lag approaches 8 hours (32 intervals of 15 minutes). At $max\_lag = 32$ (8 hours), i.e. the lag used to obtain the results in Sec. 4, the network captures $1,449$ significant links (density ≈ 0.23). Increasing the lag to 10 hours ($max\_lag = 40$) only adds a marginal number of links (total $1,541$), while, at the same time, also increasing the computational cost.

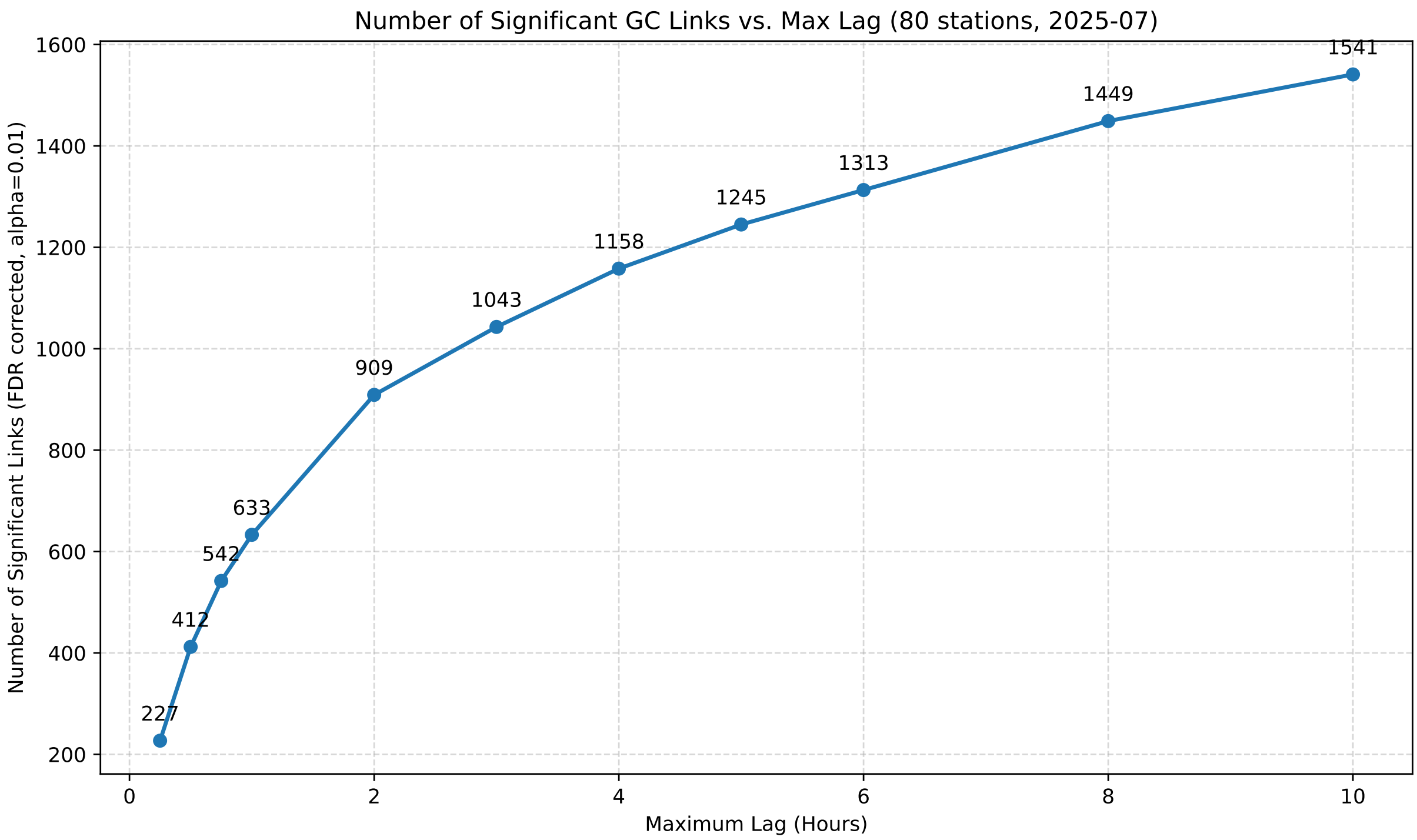

Figure 14: Sensitivity to changes in the maximum lag. The picture reports the evolution of the number of detected functional links in the SBB delay time series, as a function of the maximum lag used in the Granger Causality test.

C. Stability of the topology

The quantification and analysis of the topological metrics presented in the main text depend on multiple parameters, for which objective selection criteria are not always available. For the sake of completeness, we here analyse whether the obtained results are stable, or, on the contrary, how they are modified by different choices.

We start by considering the community structure obtained from the functional networks of arrival delays in the Swiss train system. We specifically compare three common values used for FDR thresholding, i.e. $\alpha = 0.01$, 0.05 and 0.1, as these modify the number of links retained in the network, and hence its topology. The left panel of Fig. 15 reports the Adjusted Rank Index (ARI) between pairs of values of α, i.e. an index defining how well two clustering assignments agree beyond what expected by chance. It can be appreciated that the agreement is relatively low, with an ARI between 0.27 and 0.36. This is not only due to the impact of the thresholding process, but also to the intrinsic instability of the Louvain community detection algorithm, in turn stemming from its stochastic nature. To illustrate, the right panel of Fig. 15 reports the histogram of the ARIs obtained between pairs of independent realisations of the community detection process, using the same underlying network ($\alpha = 0.01$) but different random initial seeds. The average result, of ≈ 0.45, is not substantially better than that observed in the left panel. This highlights that the obtained communities are inherently unstable and fuzzy, and that the practitioner should be careful when interpreting them. Note that this is a problem well-known in the literature, affecting both the Louvain algorithm (Traag et al., 2019) and more in general, all modularity maximisation algorithms (Good et al., 2010).

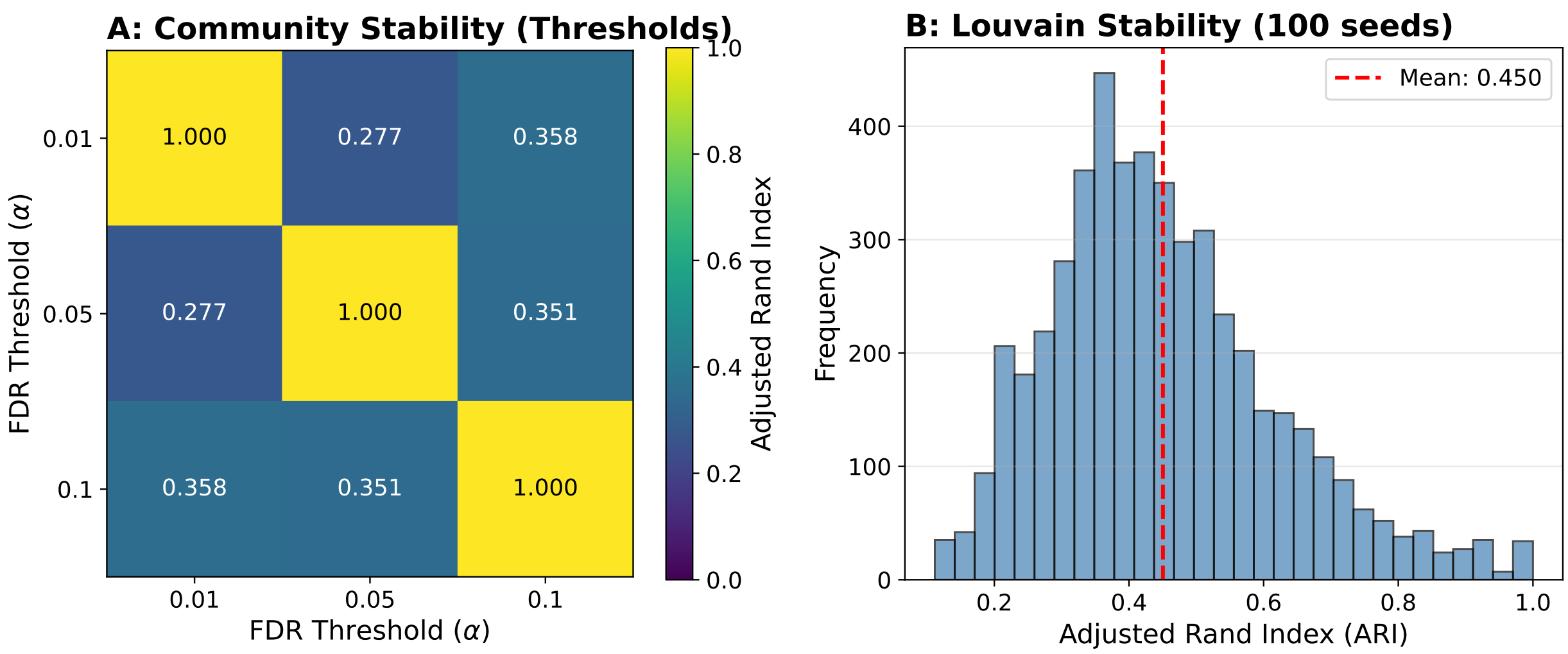

Figure 15: Stability of functional communities. (Left) Adjusted Rank Index of the community structures obtained using different values of the threshold α. (Right) Distribution of the same metric, for the communities obtained in different realisations for the same parameters and underlying functional network.

In addition to the community analysis, Table 4 reports the effect of the FDR threshold on the topology of the July 2025 functional network. As expected, relaxing the threshold from $\alpha = 0.01$ to 0.10 increases the density by approximately 47%, because weaker functional relationships are retained. The remaining raw metrics vary less over the same range: global efficiency changes by less than 14%, transitivity by less than 20%, and reciprocity by less than 27%. Thus, the number of retained links is sensitive to the threshold, while the qualitative relationships among the aggregate metrics remain consistent over the tested range. The low community agreement reported above also shows that individual Louvain assignments should not be treated as robust.

We next analyse the stability of the evolution of topological metrics through time, i.e. when these are evaluated using a rolling window approach. As in the previous case, changing the size of such window can result in different outputs. For instance, increasing its size can smooth out fast transients in the evolution; on the other hand, reducing it may result in noisier time series, potentially masking the true topological evolution. This is addressed in Fig. 16, whose panels report the evolution of three basic topological metrics, for four window sizes (see bottom legend). Beyond the natural variability of the time series, these present similar patterns irrespective of the underlying window size; for instance, all metrics present consistent changes towards the end of years 2022, 2023, and 2024. At the same time, biases

Table 4
Effect of the FDR threshold on the topological properties of the functional network (July 2025, 15-minute arrival delays, Z-score detrending, Granger causality, top-50 stations). Reciprocity, transitivity, and global efficiency are reported as raw values rather than Z-scores because the null model used for standardisation preserves the number of links, which differs at each threshold.

FDR α	Density	Links	Reciprocity	Transitivity	Efficiency
0.01	0.402	986	0.576	0.668	0.701
0.05	0.513	1257	0.676	0.745	0.757
0.10	0.591	1447	0.728	0.797	0.795

can be observed: values for transitivity (reciprocity) are consistently smaller (respectively, larger) when considering longer windows.

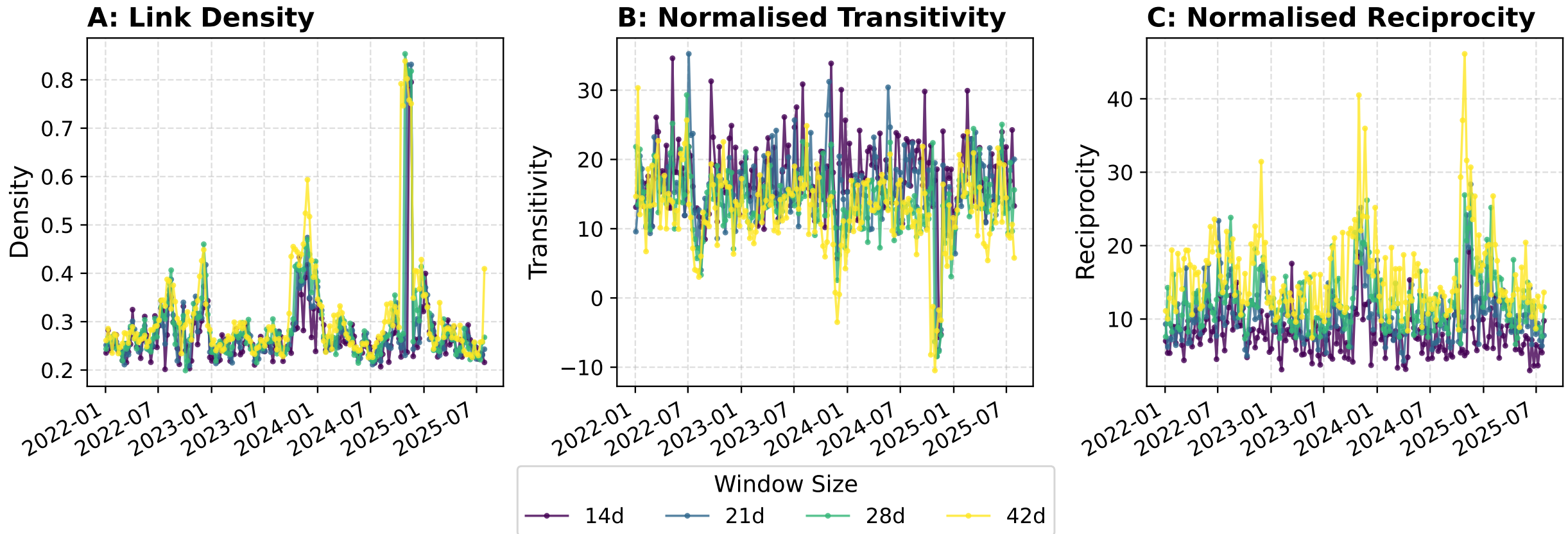

Figure 16: Stability of the evolution of topological metrics. The three panels report the evolution of the link density, transitivity, and reciprocity through time, as obtained when using sliding windows of four different sizes (see bottom legend). The transitivity and the reciprocity are reported as Z-scores relative to a random null model, and not as raw ratios (see Sec. 2.5.5).

We further evaluate sensitivity to the temporal aggregation used to construct the station-level delay time series. Figure 17 compares 5-, 15-, 30-, and 60-minute bins over 180 sliding windows per resolution. All resolutions preserve an eight-hour dependence horizon. For the 5-minute analysis, every third lag was evaluated so that its real-time lag set matches that of the 15-minute analysis without the computational cost of testing all 96 possible lags. The networks were otherwise reconstructed with daily Z-score detrending and FDR $\alpha = 0.01$. The temporal evolution of density, reciprocity, and transitivity is qualitatively similar across resolutions. Mean density is 0.346, 0.351, 0.305, and 0.229 for 5-, 15-, 30-, and 60-minute bins, respectively. The 5- and 15-minute networks therefore have similar mean density, whereas coarser aggregation produces progressively sparser networks. These observations support the use of 15-minute bins as a practical compromise between temporal detail and data sparsity.

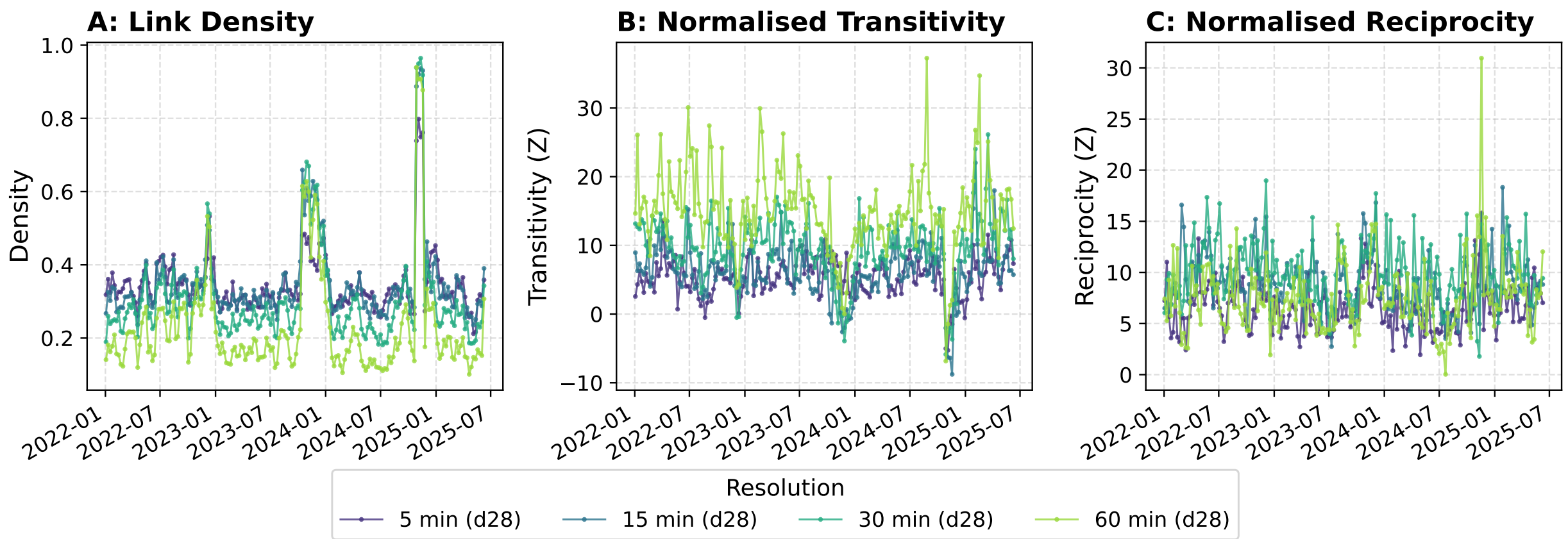

Figure 17: Sensitivity to the temporal aggregation resolution. The panels report the evolution of link density, reciprocity, and transitivity for 5-, 15-, 30-, and 60-minute arrival-delay bins over 180 28-day sliding windows. The reciprocity and the transitivity are reported as Z-scores relative to a random null model, and not as raw ratios (see Sec. 2.5.5).

D. Comparison with Physical Rail Infrastructure

The functional networks reconstructed in this study represent delay dependencies, which may or may not align with the underlying physical infrastructure. To quantify the added information provided by the functional approach, we here compare the functional delay network with the physical rail backbone of the Swiss Federal Railways (SBB). This comparison addresses whether the functional network simply mirrors the physical track topology, or, on the contrary, captures emergent operational dependencies.

D.1. Physical Network Reconstruction

We reconstructed the physical network from raw realized timetable data (*istdaten*) for July 2025. To explore different levels of operational connectivity, we consider three distinct definitions of physical adjacency:

1. **Direct Sequential (Immediate)**: An edge (A, B) exists if a train stops at station A and then immediately at station B with no intermediate stops at any station. This represents the strict track topology and the direct physical links between stations.
2. **Induced Sequential (Top-50 Nearest)**: An edge (A, B) exists if a train visits station A and then station B with no other top-50 stations in between. This represents operational adjacency within the observed system, capturing both direct track connections and sequential stops that bypass intermediate minor stations. This is the primary candidate for comparison with the functional network.
3. **Complete Trip Reachability (Full)**: An edge (A, B) exists if a train visits A and then B at any point later in the same trip, irrespective of the presence of intermediate top-50 stations. This represents both the full reachability within the long-distance network and direct mobility, i.e. pairs of stations that passengers can travel between without connecting multiple trains.

While we primarily focus on the induced network for topological comparisons, considering these three levels allows us to contextualize the functional findings within the broader reachability of the system.

D.2. Topological Distinctness and Stability

We first evaluate the structural stability of both networks using a Jaccard similarity. For each pair of physical networks, obtained using one-week data and the induced sequential approach, we calculate the Jaccard similarity; values are then averaged, and compared with those obtained in the case of the functional networks. The physical network backbone is highly static, with an average weekly similarity of $\approx 91.5\%$. In contrast, the functional network is significantly more dynamic, with a month-to-month stability of approximately 41.7% (see Fig. 18). This confirms that the functional network captures time-varying operational events and transient dependencies that are not present in the static timetable.

Furthermore, we compare the topological metrics of the physical backbone with those of the functional network (15-minute binning), as summarized in Table 5. Both networks show high non-randomness, with Z-scores for reciprocity and transitivity reaching values as high as 30–40. These high magnitudes are not numerical anomalies, but rather a direct reflection of the highly structured nature of the Swiss rail system; the functional Z-scores are, in fact, conservative relative to this physical baseline.

Table 5
Topological metrics comparison between physical (induced) and functional networks (July 2025). The reciprocity, transitivity, and (global) efficiency are reported as Z-scores relative to a random null model, as indicated by the "(Z)" label, and not as raw ratios (see Sec. 2.5.5). A high reciprocity Z-score indicates only that the number of bidirectional connections is significantly higher than in the random baseline, and does not mean that most connections in the network are bidirectional.

Network	Density	Reciprocity (Z)	Transitivity (Z)	Efficiency (Z)
Physical (Induced)	0.072	29.50	13.56	-5.60
Functional (15-minute)	0.402	11.03	4.25	-17.26

As shown in Fig. 19, the functional network occupies a distinct region in the reciprocity-density phase space. We show all three physical network definitions (direct, induced, and complete) to highlight how they vary in density and reciprocity. While the physical backbone is extremely reciprocal ($Z \approx 29.5$ for the induced case), reflecting the

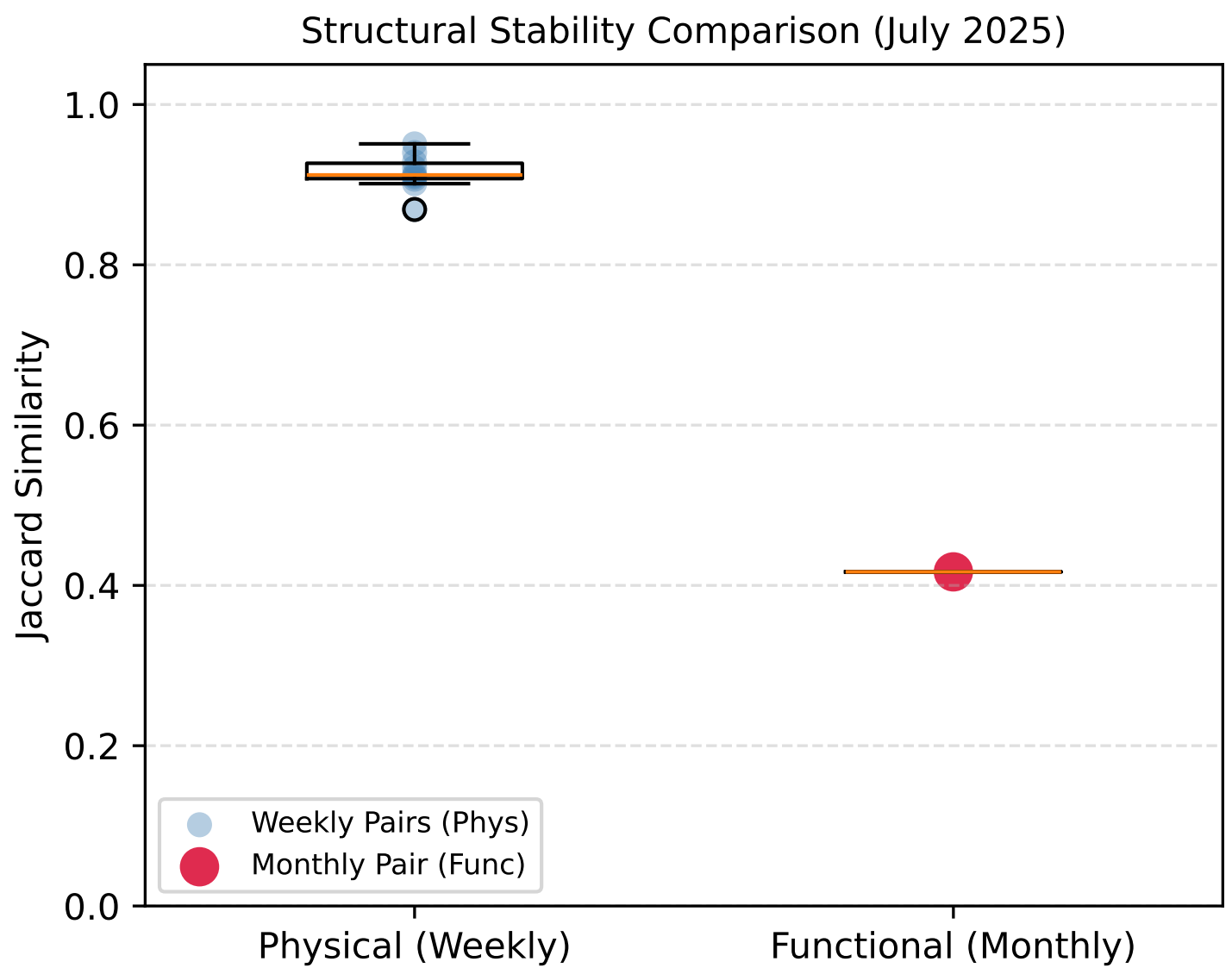

Figure 18: Structural stability of physical vs. functional networks. The box plot contrasts the high weekly stability of the physical train service backbone (induced definition) with the dynamic nature of the monthly functional delay networks.

symmetric nature of rail tracks and bidirectional services, the functional network exhibits significantly lower reciprocity ($Z \approx 11.03$). This indicates that the Granger Causality framework successfully isolates directional delay dependencies from the highly symmetric underlying physical infrastructure.

D.3. Hub Decoupling

We finally analyse the correlation between node centralities in both networks. We find that functional hubs are largely decoupled from physical ones; for instance, the Spearman rank correlation for the betweenness centrality between the induced physical and 15-minute functional networks is $\rho = -0.071$. Fig. 20 illustrates this decoupling, highlighting several "emergent functional hubs": stations that have low physical betweenness (i.e. they are not major transit junctions), but high functional importance for the propagation of delays.

D.4. Concrete Examples of Cross-Corridor Functional Links

We refine the comparison using the official Swiss GTFS timetable, which records individual train trips and their stopping patterns. For each of the 986 functional edges at $\alpha = 0.01$, we check whether the two stations are consecutive stops or share a common long-distance train service. Of these edges, 148 (15%) connect consecutive stops, while 377 (38%) connect stations that are not served by any common long-distance train. We refer to the latter as *cross-corridor* pairs and focus on them because their functional association cannot be explained by a single shared train. The 377 pairs have zero shared trips in the GTFS timetable. The timetable includes even services running only once a day or once a week, in contrast to typical IC services that run hourly or half-hourly.

Table 6 lists the ten cross-corridor pairs with the smallest GC p-values and reports their optimal lags together with the relevant GTFS routes and travel times; Figure 21 further provides a geographic and station-level overview of these relationships. These timetable data provide context for possible explanations but do not identify an operational cause. Short lags (15–60 minutes) are consistent with timetable slack (built-in buffer time that allows adjoining corridor segments to absorb small delays) or operational regulation at junctions. Intermediate lags (90–120 minutes) may reflect connection buffers at major hubs, where a guaranteed minimum transfer time allows delays from a feeder service to propagate to a connecting service on a different corridor, or rolling-stock circulation, where the same physical train set is reused across different scheduled services throughout the day. The exceptionally long lag for Rheinfelden–Romont (420 minutes) is compatible with a daily rolling-stock circulation cycle. The timetable data support these interpretations but do not directly identify the operational cause. Alternative explanations such as crew scheduling or common external disturbances cannot be excluded.

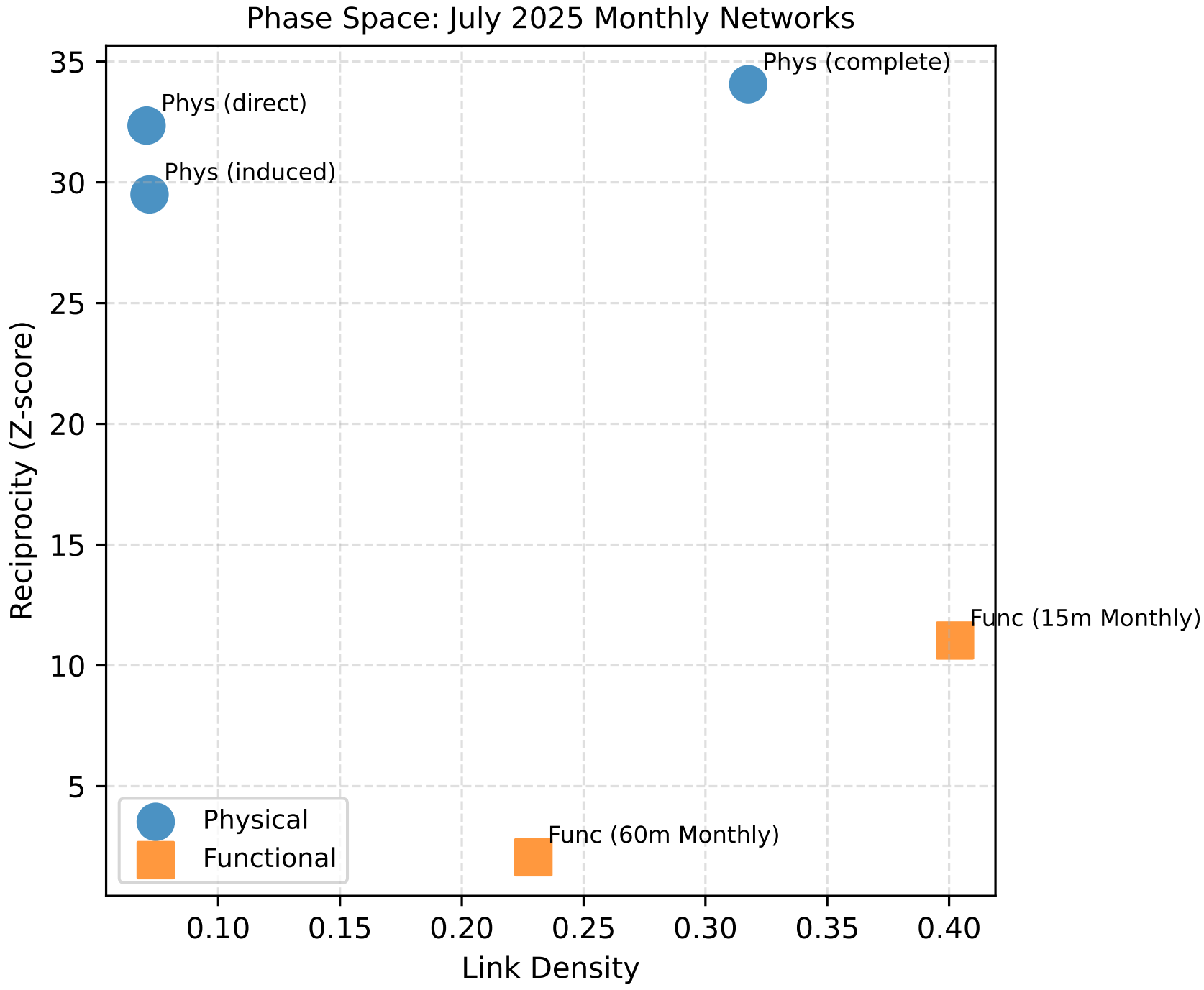

Figure 19: Reciprocity-density phase space. The functional network (15-minute) is denser and significantly less reciprocal than the physical infrastructure backbone. The reciprocity is reported as a Z-score relative to a random null model, and not as a raw ratio (see Sec. 2.5.5). The plot includes all three physical network definitions: Direct, Induced, and Complete.

Table 6
Top cross-corridor station pairs (15-minute arrival delays) with no shared direct long-distance train service in the official Swiss GTFS timetable. The table lists the top pairs from this cross-corridor group, ranked by GC p-value. GC lag is the optimal lag in minutes, distance is the geodesic distance. Legs is the minimum number of long-distance train services needed in the physical rail network. The final column reports the fastest combined route from the GTFS timetable (irregular IC services do not carry a short line number). "+N more" denotes additional multi-leg routes with the same number of legs and a travel time within twice the fastest option.

Source		Target		p-value	Lag (min)	Dist. (km)	Legs	Fastest route	
NY	Nyon	BI	Biel/Bienne	3.30e−166	90	113	2	Ir57 (49')→Yverdon: Ic5 (42')	+7 more
FCK	Frick	SIS	Sissach	8.76e−59	15	16	2	Ic (35')→Basel: Ic (17')	+1 more
BD	Baden	SIS	Sissach	8.24e−52	60	37	2	Ir16 (34')→Olten: Ic6 (16')	+3 more
SA	Sargans	TH	Thun	2.71e−39	105	142	2	Ir35 (168')→Bern: Ic8 (27')	+5 more
SA	Sargans	LST	Liestal	1.63e−37	45	139	2	Ir35 (115')→Olten: Ic6 (23')	+3 more
CH	Chur	SIO	Sion	4.63e−37	15	180	2	Ic (257')→Geneva: Ir (118')	+5 more
CH	Chur	TH	Thun	5.22e−33	120	145	2	Ir35 (190')→Bern: Ic8 (27')	+4 more
CH	Chur	LST	Liestal	4.93e−32	60	153	2	Ir35 (137')→Olten: Ic6 (23')	+4 more
AA	Aarau	FCK	Frick	2.25e−31	15	13	2	Ic8 (31')→Zürich: Ic (44')	+5 more
RF	Rheinfelden	ROM	Romont FR	5.13e−31	420	116	3	Ic (15')→Basel: Ic (63')→Luzern: Ic (105')	+28 more

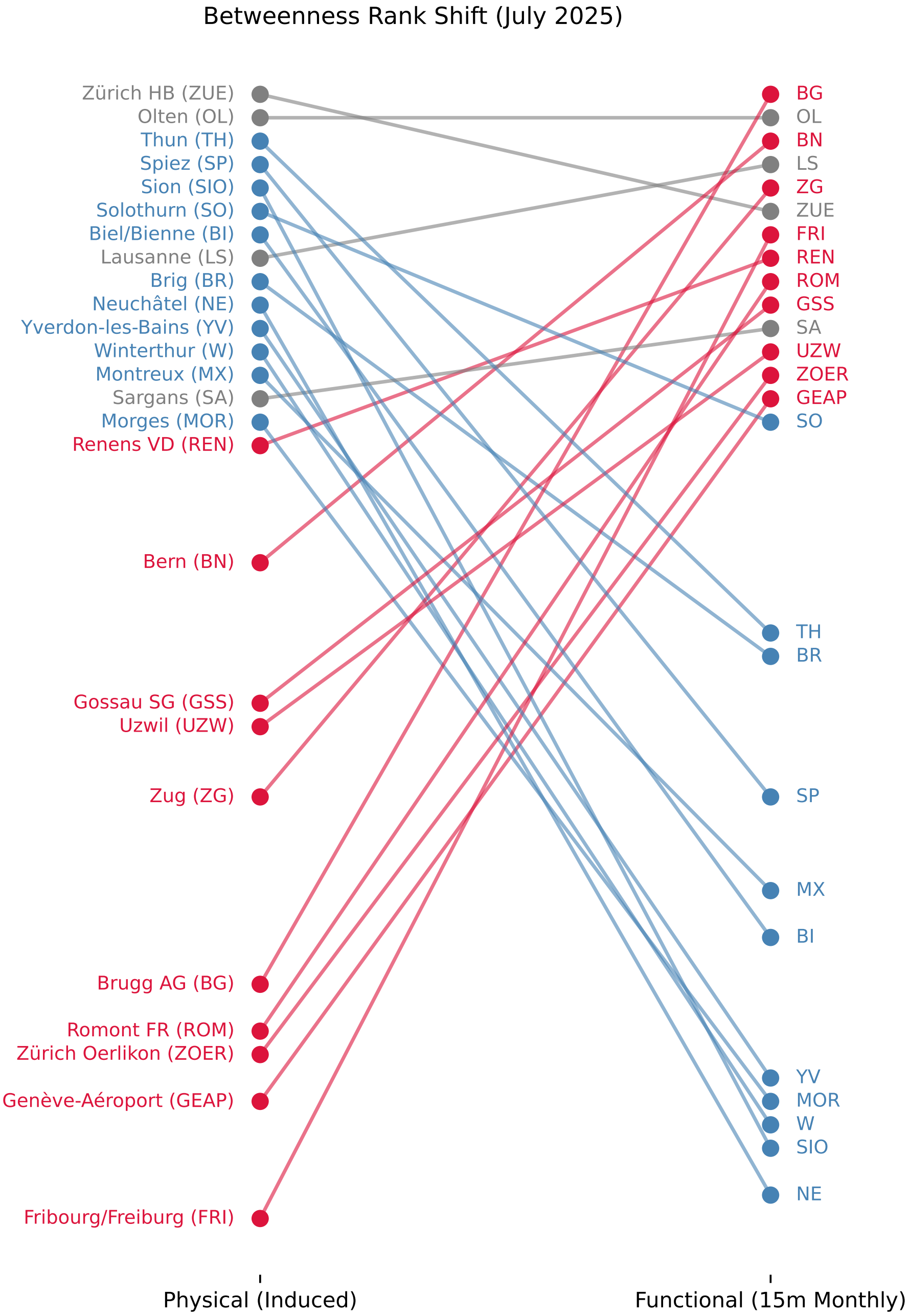

Figure 20: Centrality rank shifts. The slopegraph compares node ranks based on betweenness centrality in the induced physical (left) and 15-minute functional (right) networks. Stations are coloured based on their rank shift: red indicates emergent functional hubs, where the functional rank is significantly higher than the physical one (shift > 5); blue indicates stations where the physical rank is significantly higher (shift < -5); and gray indicates stations with stable ranks ($|\text{shift}| \leq 5$). These results confirm that functional connectivity provides added explanatory power, identifying operational bottlenecks that cannot be inferred from the physical topology alone.

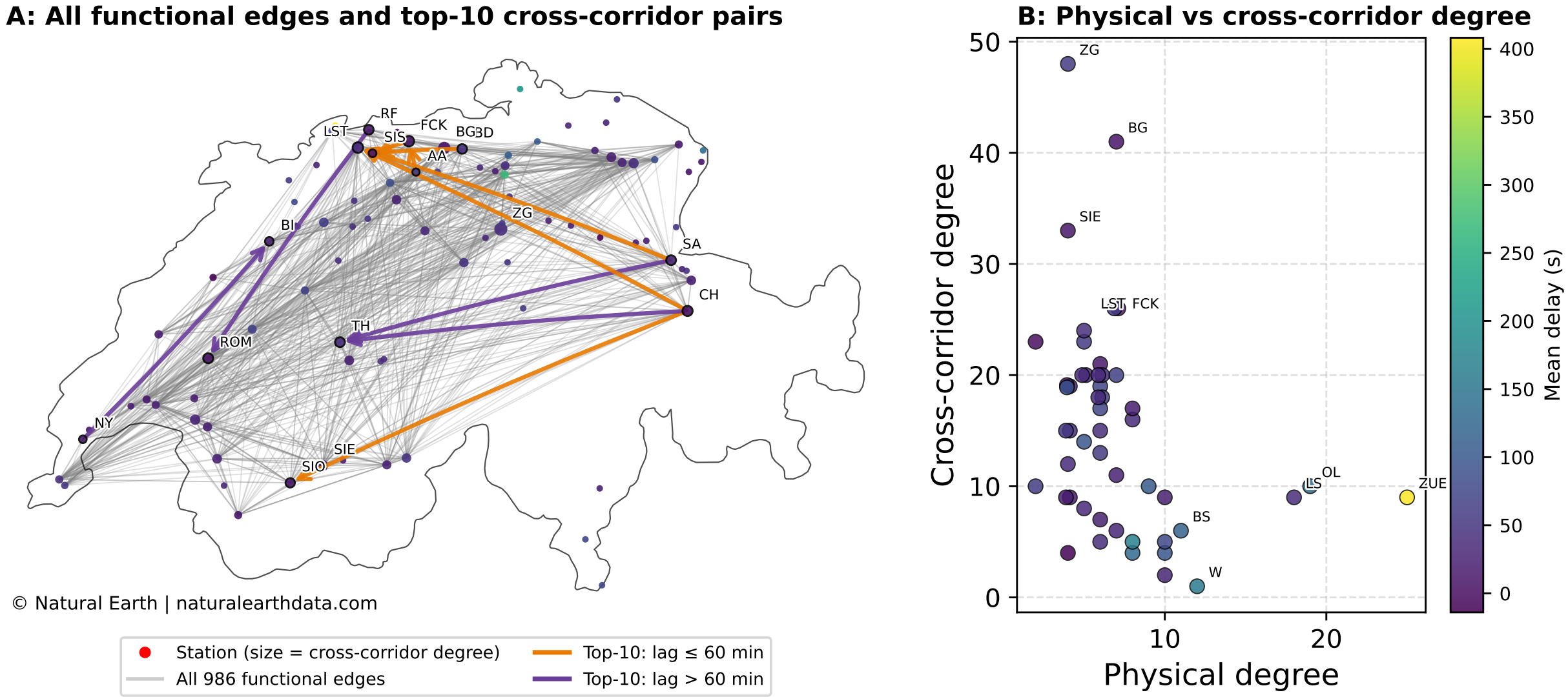

Figure 21: Cross-corridor functional connectivity in July 2025. The geographic panels show the functional network and highlight the ten station pairs with the smallest GC p-values among those without a shared long-distance train service, coloured by their optimal GC lag. The station-level panel compares physical and cross-corridor degree, with colour indicating mean arrival delay.
